# *Fusarium* mycotoxins: The major food contaminants

**DOI:** 10.1002/mlf2.12112

**Published:** 2024-05-13

**Authors:** Zheng Qu, Xianfeng Ren, Zhaolin Du, Jie Hou, Ye Li, Yanpo Yao, Yi An

**Affiliations:** ^1^ Agro‐Environmental Protection Institute Ministry of Agriculture and Rural Affairs Tianjin China; ^2^ Institute of Quality Standard and Testing Technology for Agro‐Products Shandong Academy of Agricultural Sciences Jinan China

**Keywords:** climate change, *Fusarium* mycotoxin, management strategy, mycotoxin detection

## Abstract

Mycotoxins, which are secondary metabolites produced by toxicogenic fungi, are natural food toxins that cause acute and chronic adverse reactions in humans and animals. The genus *Fusarium* is one of three major genera of mycotoxin‐producing fungi. Trichothecenes, fumonisins, and zearalenone are the major *Fusarium* mycotoxins that occur worldwide. *Fusarium* mycotoxins have the potential to infiltrate the human food chain via contamination during crop production and food processing, eventually threatening human health. The occurrence and development of *Fusarium* mycotoxin contamination will change with climate change, especially with variations in temperature, precipitation, and carbon dioxide concentration. To address these challenges, researchers have built a series of effective models to forecast the occurrence of *Fusarium* mycotoxins and provide guidance for crop production. *Fusarium* mycotoxins frequently exist in food products at extremely low levels, thus necessitating the development of highly sensitive and reliable detection techniques. Numerous successful detection methods have been developed to meet the requirements of various situations, and an increasing number of methods are moving toward high‐throughput features. Although *Fusarium* mycotoxins cannot be completely eliminated, numerous agronomic, chemical, physical, and biological methods can lower *Fusarium* mycotoxin contamination to safe levels during the preharvest and postharvest stages. These theoretical innovations and technological advances have the potential to facilitate the development of comprehensive strategies for effectively managing *Fusarium* mycotoxin contamination in the future.

## INTRODUCTION

Fungi are a kind of eukaryote with a vegetative structure that can be filamentous or unicellular. Their cell walls are composed of chitin, chitosan, or polysaccharides, and they reproduce by spores that are produced asexually or sexually. Fungi are ubiquitous in the biosphere and the second most species‐rich eukaryotic organisms after insects^1^. They play a pivotal role in the intricate processes of the nutrient cycle and ecosystem balance and are consumed as food and used in the production of industrial materials^2^. Similar to viruses and bacteria, fungi also have negative effects on humans. More than 500 fungal species are capable of infecting the human body, which causes at least 1.5 million deaths globally each year^3^. In plantation crop production, plant pathogenic fungi can cause economic losses of upward of hundreds of billions of dollars each year. Plant pathogenic fungi adversely affect crop growth, yield, and quality, and mycotoxin contamination is one of the most important causes of quality degradation.^4^

Mycotoxins are natural toxic compounds with low molecular weight (often less than 1000 Da) that are synthesized by fungi and have the characteristics of nephrotoxicity, hepatotoxicity, carcinogenicity, teratogenicity, immune toxicity, neurotoxicity, genotoxicity, mutagenicity, cytotoxicity, reproductive toxicity, alimentary canal toxicity, dermal toxicity, and so on^5^, ^6^. Mycotoxin exposure, whether in humans or in animals, even at low concentrations, can lead to acute or chronic diseases and, in certain instances, death. Cereals, nuts, fruits, spices, legumes, and their products or byproducts are most likely to contain mycotoxins. Based on a previous estimation by the Food and Agriculture Organization (FAO) of the United Nations, approximately 25% of cereals produced worldwide are contaminated by mycotoxins^7^. However, a recent study conducted by Eskola et al. revealed that the contamination rate could be 60%–80%^8^. Mycotoxins enter the food chain via fungal infections of crops, which can occur at any phase of crop production, including planting, harvesting, and storage. Mycotoxins can be introduced into the human body directly via the ingestion of contaminated foods or derived products, or indirectly through the consumption of eggs, edible offal, meat, milk, and related products from livestock that consume contaminated feed^8^.

Mycotoxins are not essential to the growth or development of fungi, but they seem to be a way for fungi to reduce the number of superfluous precursors^9^. It has also been suggested that mycotoxins contribute to the defensive tactics of mycotoxigenic fungi against other microorganisms. Moreover, mycotoxins exert a substantial influence on the pathogenicity, aggressiveness, and virulence of mycotoxigenic fungi^10^. More than 400 varieties of mycotoxins have been identified, but aflatoxin (AF), ochratoxins, fumonisins (FUMs), trichothecenes (TRIs), patulin, citrinin (CIT), and zearalenone (ZEA) are those most inextricably linked to agriculture, economics, and public health^5^, ^6^. There are three dominant toxigenic fungal genera: *Aspergillus*, *Fusarium*, and *Penicillium* ^6^. *Fusarium* species usually infect crops and produce mycotoxins before or immediately after harvest, while the *Aspergillus* and *Penicillium* species are more commonly associated with foods during drying and storage^5^.

**Figure 1:**
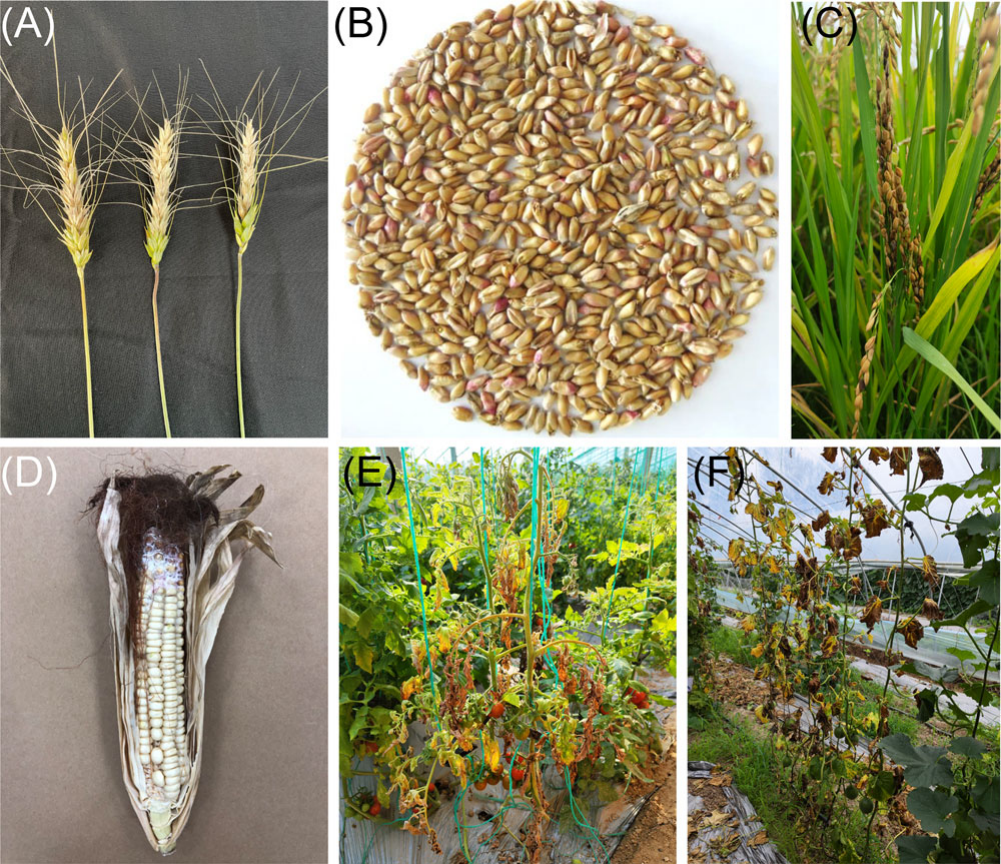
Examples of plant *Fusarium* diseases. (A) Fusarium head blight (FHB) of wheat caused by *Fusarium graminearum* (photo credit: Dr. Jie Wang). (B) Diseased wheat kernels due to FHB (photo credit: Dr. Binnian Tian). (C) Rice spikelet rot disease caused by *Fusarium proliferatum* (photo credit: Dr. Yanpo Yao). (D) Fusarium ear rot of corn caused by *Fusarium verticillioides* (photo credit: Dr. Zheng Qu). (E) Fusarium wilt of cherry tomato caused by *Fusarium oxysporum* (photo credit: Dr. Ying Zhao). (F) Fusarium wilt of melon caused by *F. oxysporum* (photo credit: Dr. Ying Zhao).

**Figure 2:**
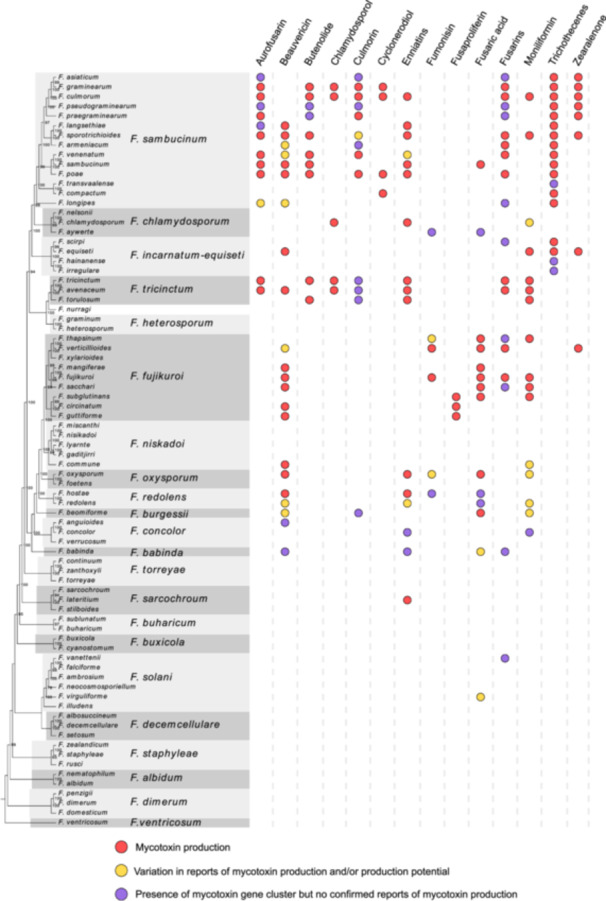
The varieties of mycotoxin production in *Fusarium* species. On the left side, the cladogram depicts phylogenetic relationships among the chosen set of *Fusarium* species. On the right side, the circle represents the mycotoxin production abilities of *Fusarium* species. The cladogram was constructed by maximum likelihood analysis using the *RPB1* + *RPB2* dataset described by O'Donnell et al.^16^ in RAxML 8.2.10. Support for branches was determined through bootstrap analysis (1000 replicates), and only values of more than 60 are shown. The distribution of mycotoxin production was based on the work of Munkvold et al.^11^. White space indicates that there are no chemical or genetic evidence for mycotoxin production.

*Fusarium*, a prominent genus of plant pathogenic and mycotoxin‐producing fungi worldwide, targets various plant parts, including grains, seedlings, heads, roots, and stems (Figure 1), causing yield loss and quality reduction of crops^11^. For example, Fusarium head blight (FHB) is a highly destructive disease that affects cereals and results in both significant yield reduction and a negative influence on grain quality^12^. Currently, *Fusarium* comprises more than 300 phylogenetically distinct species and has been classified into 23 informal species complexes^13^. Different *Fusarium* species have various abilities to produce mycotoxins. The *Fusarium sambucinum* species complex includes many devastating plant pathogens, most of which are important mycotoxin producers (Figure 2); the three predominant classes of mycotoxins synthesized by *Fusarium* species are FUMs, TRIs, and ZEA. Moreover, a type of TRI analog, deoxynivalenol (DON), is consistently regarded as a significant concern in the realm of food safety due to its prevalent occurrence as a grain contaminant. Due to a lack of adequate understanding, other toxic *Fusarium* metabolites, which are commonly referred to as emerging mycotoxins and include beauvericin (BEA), enniatins, fusaproliferin, fusaric acid, fusarins, and moniliformin (MON), neither undergo routine determination nor are subject to legislative regulation^11^. Furthermore, *Fusarium* mycotoxins can be converted into modified forms, known as modified mycotoxins, by plants, microorganisms, and chemical or physical approaches during processing, such as acid, alkali, heat, pressure, and irradiation. The modified mycotoxins can coexist with their parent forms^14^. Among these, the modified mycotoxins generated as a result of plant defense mechanisms, primarily via glucosylation catalyzed by uridine diphosphate‐glucosyltransferases, are known as masked mycotoxins^15^. The glucose conjugates of mycotoxins, the most commonly identified masked mycotoxins in current research, include DON‐3‐glucoside (DON‐3G), T‐2‐toxin‐3‐glucoside, HT‐2‐toxin‐3‐glucoside, nivalenol‐3‐glucoside (NIV‐3G), ZEA‐14G, α‐zearalenol‐14‐glucoside (α‐ZEL‐14‐G), and β‐ZEL‐14‐G. Although the limited available information suggests that masked mycotoxins show lower toxicity than their parent forms, the free forms of masked mycotoxins can still cause unpredicted toxicity via hydrolysis by mammalian gut microorganisms^15^.

In this review, we outline the main types of *Fusarium* mycotoxins and elucidate their impact on food contamination as well as associated hazards. We then review the interactions between climate change (CC) and *Fusarium* mycotoxin risks. Finally, we focus on the detection technologies and management strategies for *Fusarium* mycotoxin contamination. This review provides a reliable reference source for *Fusarium* mycotoxin control.

## MAIN *FUSARIUM* MYCOTOXINS

### TRIs

**Figure 3:**
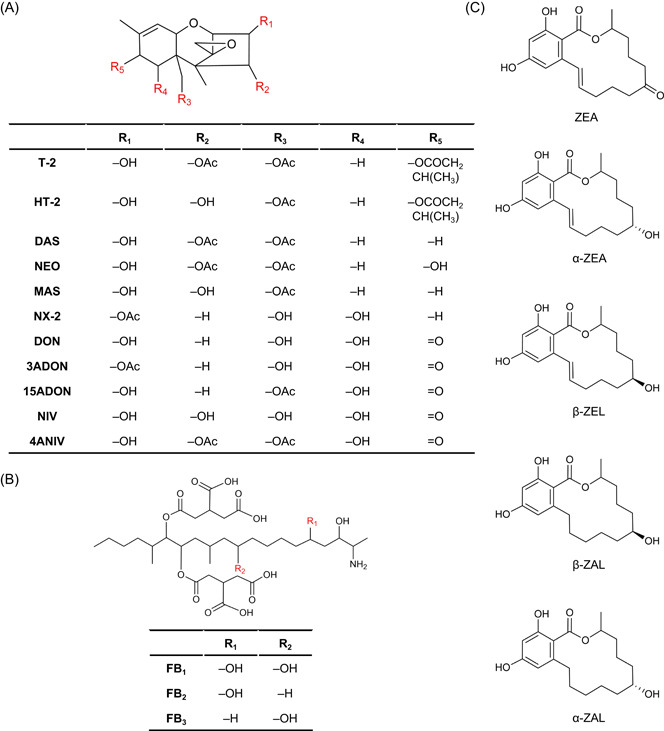
Chemical structures of primary *Fusarium* mycotoxins. (A) Chemical structures of Types A and B trichothecenes produced by *Fusarium*, such as diacetoxyscirpenol (DAS), deoxynivalenol (DON), HT‐2 toxin (HT‐2), T‐2 toxin (T‐2), monoacetoxyscirpenol (MAS), neosolaniol (NEO), and nivalenol (NIV), the acetylated derivatives of DON and NIV (3ADON, 15ADON, 4ANIV), and the new trichothecene mycotoxin NX‐2. (B) Chemical structures of B‐series fumonisins, which show structural variations based on the presence or absence of hydroxyl groups at C‐5 (R_1_) and C‐10 (R_2_). (C) Chemical structure of zearalenone and its derivatives.

The most extensive and economically important cluster of *Fusarium* mycotoxins are the TRIs, which include over 200 compounds. TRIs are tetracyclic sesquiterpenoid substances characterized by a single six‐membered ring with a single oxygen atom flanked by two carbon rings (Figure 3A). This core structure shows a double bond between C‐9 and C‐10, along with an epoxide ring at the C‐12 and C‐13 positions^17^, ^18^, ^19^. According to the various patterns of oxygenation and esterification at the C‐3, C‐4, C‐7, C‐8, and C‐15 positions, TRIs can be classified into four groups: Types A, B, C, and D. *Fusarium* species produce Type A and Type B TRIs, which are concentrated in the *F. sambucinum* and *Fusarium incarnatum*‐*equiseti* species complexes, respectively^11^, ^19^. Representative Type A TRIs include T‐2 toxin (T‐2), HT‐2 toxin (HT‐2), neosolaniol (NEO), diacetoxyscirpenol (DAS), monoacetoxyscirpenol (MAS), and NX‐2. Type B TRIs are represented by DON, NIV and their acetyl derivatives: 3‐acetyldeoxynivalenol (3ADON), 15‐acetyldeoxynivalenol (15ADON), and 4‐acetylnivalenol (4ANIV). The toxic effects of TRIs can be teratogenic, nephrotoxic, hepatotoxic, cytotoxic, alimentary canal toxic, genotoxic, and immune toxic^20^.

**Figure 4:**
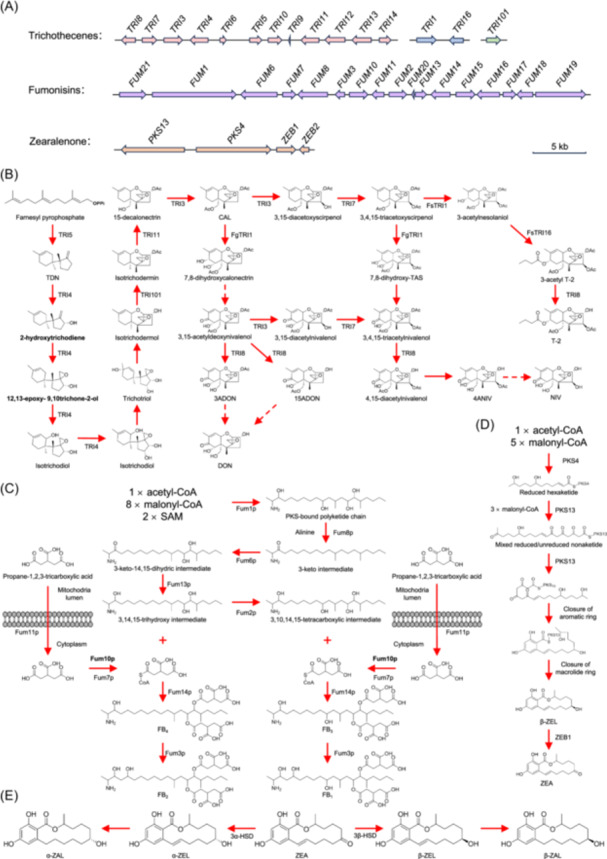
The formation approaches of *Fusarium* mycotoxins. (A) Mycotoxin biosynthetic genes and gene clusters in *Fusarium*. Arrows denote the location and transcriptional orientation of genes, while the corresponding gene name is provided adjacent to each arrow. (B) The proposed pathway of TRI biosynthesis. The 3,4,15‐triacetoxyscirpenol is an important branching point. One path leads to a Type A TRI, and the other leads to a Type B TRI. In *F. sporotrichioides*, FsRI1 exclusively catalyzes hydroxylation of 3,4,15‐triacetoxyscirpenol at C‐8, resulting in Type A TRIs, while in *F. graminearum*, FgTRI1 mediates hydroxylation of 3,4,15‐triacetoxyscirpenol at both C‐7 and C‐8, resulting in Type B TRIs. Dashed arrows denote steps where gene assignment has not been established. (C) The proposed pathway of FUM biosynthesis. Fum11p, a tricarboxylate transporter, transports tricarboxylic acid precursors out of the inner mitochondrial lumen for FUM biosynthesis, while Fum7p and Fum10p catalyze the conversion of tricarboxylic acid precursors into acetyl CoA‐activated tricarballylic acid. This product is then esterified to the polyketide backbone at C‐14 and C‐15 by Fum14p to produce either FB_3_ or FB_4_. Finally, the hydroxylation of FB_3_ and FB_4_ by Fum3p yields FB_1_ and FB_2_, respectively. (D) The proposed pathway of ZEA biosynthesis. The biosynthesis begins with PKS4, which facilitates the condensation of carbons derived from one acetyl‐CoA and five malonyl‐CoAs. PKS13 completes the polyketide backbone as an extender unit and is responsible for cyclization and aromatization. As the final step, β‐ZEL is converted into ZEA by ZEB1. (E) The formation pattern of metabolites of ZEA. The C6 keto group of ZEA is reduced to yield α‐ZEL and β‐ZEL, followed by a subsequent reduction at the C11–C12 double bond that results in the formation of α‐ZAL and β‐ZAL, respectively. Those proposed biosynthesis and metabolites pathways were derived from the works of McCormick et al.^17^, Alexander et al^21^, Nahle et al^23^ and EFSA Panel on Contaminants in the Food Chain (CONTAM) et al^74^. CAL, calonectrin; FgTRI1, *F. graminearum* TRI1; FsTRI1, *F. sporotrichioides* TRI1; FUM, fumonisin; PKS, polyketide synthase; SAM, S‐adenosyl methionine; TRI, trichothecenes; ZEA, zearalenone.

In *Fusarium*, TRI biosynthetic enzymes are encoded by a total of 15 *TRI* genes, which are distributed across three different loci on different chromosomes: the single‐gene *TRI101* locus, the two‐gene *TRI1*‐*TRI16* locus, and the 12‐gene core *TRI* cluster (Figure 4A)^21^, ^22^. In *F. graminearum*, the *TRI16* homolog is rendered nonfunctional as a result of multiple insertions and deletions within its coding region. Table 1 shows the phenotypes of mutants with *TRI* gene disruption or deletion in *F. sporotrichioides*. TRI5 catalyzes the cyclization of farnesyl pyrophosphate (FPP) into trichodiene (TDN), which represents the initial enzymatic step in TRI biosynthesis (Figure 4B). TDN is subsequently transformed into calonectrin (CAL) by TRI4, TRI101, TRI11, and TRI3. The catalytic reactions from FPP to CAL are conserved across *Fusarium* species that synthesize Type A and Type B TRIs. The allelic variants of TRI1 are responsible for the significant structural disparities between Type A and Type B TRIs. In *F. sporotrichioides*, TRI1 exclusively catalyzes hydroxylation at C‐8, resulting in Type A TRIs, while *F. graminearum* TRI1 mediates hydroxylation at both C‐7 and C‐8, resulting in Type B TRIs^17^, ^21^, ^22^.

**Table 1:** Function of *Fusarium* mycotoxin biosynthesis genes and phenotypes of their mutants.

Mycotoxin	Gene	Predicted function	Mutant phenotype
TRIs	*TRI8*	TRI‐3‐*O*‐esterase	3‐acetyl T‐2, TAS
	*TRI7*	TRI‐4‐*O*‐acetyltransferase	HT‐2
	*TRI3*	TRI‐15‐*O*‐acetyltransferase	15‐decalonectrin, 3,15‐didecalonectrin
	*TRI4*	Trichodiene oxygenase	Trichodiene
	*TRI6*	Transcription factor	Low levels of trichodiene
	*TRI5*	Trichodiene synthase	No TRIs
	*TRI10*	Regulatory gene	No TRIs
	*TRI9*	Unknown	Not determined.
	*TRI11*	Isotrichodermin 15‐oxygenase	Isotrichodermin
	*TRI12*	TRI efflux pump	No TRIs
	*TRI13*	Calonectrin 4‐oxygenase	4‐deoxy T‐2 toxin, 8‐hydroxycalonectrin, 8‐hydroxy‐3‐decalonectrin
	*TRI14*	Virulence factor	T‐2 toxin
	*TRI1*	C‐8 or C‐7,8 oxygenase	4,15‐DAS
	*TRI16*	C‐8 acyltransferase	Neosolaniol
	*TRI101*	C‐3 acyltransferase	Isotrichodermol
FUMs	*FUM21*	Cys‐6 transcription factor	No FUMs
	*FUM1*	PKS	No FUMs
	*FUM6*	Cytochrome P450 monooxygenase and reductase	No FUMs
	*FUM7*	Alcohol dehydrogenase	Tetradehydro‐FB
	*FUM8*	α‐Oxoamine synthase	No FUMs
	*FUM3*	Dioxygenase	FB_2_, FB_4_
	*FUM10*	Acyl‐CoA synthetase/acyl‐protein synthetase	Hydrolyzed FB_3_, hydrolyzed FB_4_
	*FUM11*	Tricarboxyllic acid transporter	FB_1_, FB_2_, FB_3_, FB_4_
			Half‐hydrolyzed FB_1_, FB_2_, FB_3_, FB_4_
			Keto half‐hydrolyzed FB_1_, FB_2_, FB_3_, FB_4_
	*FUM2*	Cytochrome P450 monooxygenase	FB_2_, FB_4_
	*FUM20*	Unknown	Not determined
	*FUM13*	Short‐chain dehydrogenase/reductase	3‐keto FB_3_, 3‐keto FB_4_
	*FUM14*	Nonribosomal peptide synthase (peptidyl and condensation domains)	Hydrolyzed FB_3,_ hydrolyzed FB_4_
	*FUM15*	Cytochrome P450 monooxygenase	No effect
	*FUM16*	Acyl‐CoA synthetase/acyl‐protein synthetase	No effect
	*FUM17*	Ceramide synthase	No effect
	*FUM18*	Ceramide synthase	No effect
	*FUM19*	ABC transporter	Increased ratio FB_1_:FB_3_
ZEA	*PKS13*	PKS	No ZEA
	*PKS4*	PKS	No ZEA
	*ZEB1*	Alcohol oxidase	β‐ZEL
	*ZEB2*	Transcription factor	No ZEA

The production profiles of *Fusarium* mycotoxins were determined by liquid chromatography–mass spectrometry analysis of extracts and filtrates of *Fusarium* mycotoxin biosynthesis gene mutants. FUMs, fumonisins; PKS, polyketide synthase; TAS, 3,4,15‐triacetoxyscirpenol; TRIs, trichothecenes; ZEA, zearalenone. Data were extracted from Alexander and colleagues^21^, ^23^, ^24^.

DON (also called vomitoxin) is one of the mycotoxins linked to FHB, and it is synthesized in *F. graminearum, F. culmorum*, and so on. The formal chemical name of DON is 3α,7α,15‐trihydroxy‐12,13‐epoxymonospora‐9‐ene‐8‐one^25^. It mainly contaminates cereal crops, such as maize, wheat, rice, and barley, as well as some cash crops. All over the world, DON contamination causes economic losses of billions of dollars each year. The Panel on Contaminants in the Food Chain, which is part of the European Food Safety Authority, examined 26,613 cereal samples from 21 European countries and found that the DON contamination rate was close to 50%^26^. Yan et al. collected 579 wheat samples and 606 maize samples from the major wheat‐ and maize‐producing provinces in China and determined the co‐occurrence of type‐B TRIs; all of the wheat samples showed positive results for DON, whereas 99.83% of the maize samples were DON‐positive^27^. In humans and animals, DON typically causes diarrhea, vomiting, and gastrointestinal inflammation. The chronic consumption caused by DON can result in immune‐suppressive diseases, growth impairment, and abnormalities of the reproduction and nervous systems^28^. The FAO and World Health Organization (WHO) first identified DON as a highly hazardous food contaminant in the 1970s^25^.

**Figure 5:**
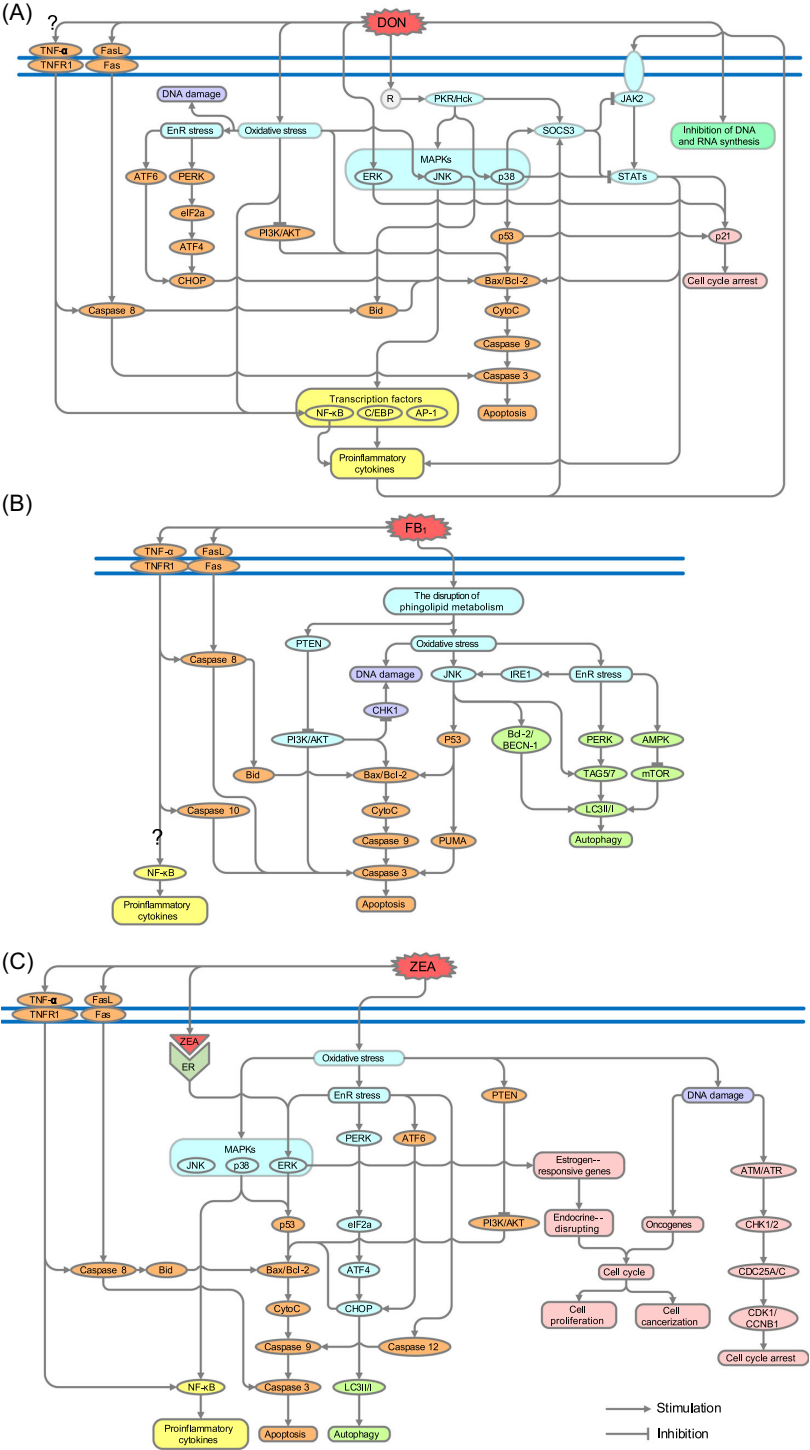
The toxicological mechanisms of the main *Fusarium* mycotoxins. (A) DON‐induced oxidative stress can cause DNA damage, EnR stress, and the suppression of the PI3K/AKT pathway. Consequently, it can lead to a decrease in *Bcl‐2* gene expression, an increase in *Bax* gene expression, and elevated levels of *CytoC* expression. It enhances the upregulated expression of Caspase 9 and Caspase 3 and induces apoptosis. DON can efficiently induce the activation of MAPKs through a mechanism referred to as the “ribotoxic stress response” and hijack the JAK2/STAT‐3 pathway to induce apoptosis, proinflammatory cytokine upregulation, and cell cycle arrest. DON also activates Caspase 8 to increase expression of Caspase 3 by the tumor necrosis factor‐α and Fas/FasL signaling pathways, leading to apoptosis. (B) FB_1_ disrupts sphingolipid metabolism, causing DNA damage and apoptosis through the PTEN/PI3K/AKT signaling pathway as well as oxidative stress, which further entrenches DNA damage and apoptosis and induces EnR stress. FB_1_‐induced EnR stress not only causes apoptosis by the JNK/p53/PUMA/Caspase 3 pathway but also leads to autophagy via signaling pathways mediated by IRE1, PERK, and AMPK. IRE1 can activate JNK, which results in the subsequent elevation of BECN1, ATG5, and ATG7 and the conversion of LC3‐I. PERK induces the expression of ATG5 and ATG7 to form the ATG5–ATG7 complex and promotes the conversion of LC3‐I into LC3‐II. Under EnR stress, AMPK inhibits the anabolic process of mTOR, thereby increasing the expression of autophagy‐related genes and triggering autophagy. (C) ZEA and its derivatives can bind to ERs to elicit estrogen‐like effects and promote cellular proliferation. ZEA‐induced DNA damage can upregulate ATM and ATR expression and activate DNA damage checkpoints CHK1 and CHK2 to repair DNA damage. Next, the expression levels of CDC25A and CDC25C are upregulated, which promotes the expression of CCNB1 and CDK1, thus preventing the cell cycle from exiting the G2/M phase. Moreover, ZEA can induce apoptosis and autophagy similar to FB_1_ and DON. Question marks indicate indeterminate roles. EnR, endoplasmic reticulum; MAPK, mitogen‐activated protein kinase.

By binding to the 60S ribosomal subunit, DON can disrupt the action of peptidyl transferase and inhibit protein synthesis^18^, ^28^. Moreover, DON can modulate mitogen‐activated protein kinase (MAPK) activity, which includes extracellular signal‐regulated kinase (ERK), c‐Jun N‐terminal kinase (JNK), and p38 mitogen‐activated protein kinase (p38), via the “ribotoxic stress response” by rapidly activating double‐stranded RNA‐associated protein kinase (PKR) and hematopoietic cell kinase (Hck^29^; Figure 5A). DON‐induced MAPK activation can mediate the upregulation of transcription factors (nuclear factor kappa‐light‐chain‐enhancer of activated B cells [NF‐κB], activating protein 1 [AP‐1], and CCAAT/enhancer‐binding protein [C/EBP]) to promote the expression of proinflammatory cytokines, such as interleukin‐6 (IL‐6), IL‐1β, and tumor necrosis factor‐α (TNF‐α), which cause inflammation^28^, ^30^. DON causes oxidative stress by inhibiting the expression of glutathione (GSH), superoxide dismutase (SOD), catalase (CAT), and GSH peroxidase (GSH‐PX), which engage in the repair of oxidative stress‐induced damage, decreasing total antioxidant capacity and glutathione S‐transferase levels^31^. However, DON can also induce nuclear factor‐erythroid 2‐related factor 2 (Nrf2) and heme oxygenase‐1 (HO‐1) activation to remove excessive reactive oxygen species (ROS). These results show that DON not only elicits oxidative stress but also impedes its occurrence. The potential impact of DON may vary depending on the dosage, although the precise mechanism remains incompletely elucidated^32^.

The relationship between oxidative stress and inflammation is interrelated and interdependent. Specifically, the products of oxidative stress (primarily ROS) have been shown to increase proinflammatory responses. Additionally, inflammatory cells release significant amounts of ROS at sites of inflammation, exacerbating oxidative damage^33^. In most cell types, mitochondria are not only the largest contributors to intracellular ROS production but also the targets of cellular ROS. A feed‐forward detrimental cycle exists between the generation of ROS and mitochondrial damage, wherein ROS‐damaged mitochondria undergo dysfunctionality, subsequently leading to an exacerbation in intracellular ROS production^34^. In the case of oxidative stress, damaged mitochondria show a decrease in mitochondrial membrane potential, which is closely related to apoptosis^28^, ^32^. The B‐cell lymphoma 2 (*Bcl‐2*) gene family regulates apoptosis through the mitochondrial pathway. Among this gene family, the Bcl‐2‐associated X (*Bax*) gene and Bcl‐2 antagonist/killer‐1 (*Bak‐1*) gene are important proapoptotic genes, while *Bcl‐2* is an antiapoptotic gene. The augmented Bax/Bcl‐2 ratio serves as a pivotal indicator of the initiation of apoptosis. Caspases (cysteine‐aspartic proteases, cysteine aspartases, or cysteine‐dependent aspartate‐directed proteases), a group of protease enzymes, are crucial players in the process of apoptosis, with Caspase 3 being known as the executor of apoptosis^35^. DON‐induced oxidative stress results in oxidation and damage of DNA and lipids through the elevation of ROS levels, finally leading to apoptosis through the mitochondrial pathway via activation of the JNK pathway, which increases the Bax/Bcl‐2 ratio and induces the conversion of Caspase 9 into Caspase 3. In addition, this process triggers proinflammatory cytokine expression by upregulating NF‐κB^32^, ^36^, ^37^. Moreover, DON can also induce apoptosis by regulating the Janus kinase 2/signal transducers and activators of transcription (JAK2/STAT) pathway and inhibiting the phosphatidylinositol 3‐kinase/threonine kinase (PI3K/AKT) pathway, the latter of which is important in inhibiting apoptosis because it regulates downstream effector molecules (e.g., suppressing Bax translocation)^36^, ^38^, ^39^, ^40^. In addition, DON can disturb normal cell cycle progression by upregulating ERK and JAK2/STAT to promote cyclin‐dependent kinase inhibitor p21 expression^40^. Perturbations in endoplasmic reticulum (EnR) function resulting from enhanced protein synthesis or the accumulation of misfolded proteins give rise to a condition known as EnR stress^41^. The generation of ROS can elicit an elevation in the EnR stress level, while EnR stress can induce the production of ROS in the EnR and mitochondria^42^. EnR stress can induce cellular death, including apoptosis and autophagy, through three classical signaling pathways (protein kinase RNA‐like endoplasmic reticulum kinase [PERK], activating transcription factor 6 [ATF6], and the inositol‐requiring protein 1 [IRE1]‐mediated signaling pathway). In EnR stress‐induced apoptosis, the C/EBP homologous protein (CHOP) transcription factor plays a pivotal role in the PERK, ATF6, and IRE1 signaling pathways^43^. To date, studies have demonstrated only that 3ADON has the capability to induce apoptosis in a mouse liver by inducing EnR stress via the IRE1 pathway^44^. No similar study of DON yet exists. The DON‐activated death receptor pathway of apoptosis includes the TNF‐induced model and the Fas/FasL‐mediated model (Fas, the first apoptosis signal, is also known as Apo‐1 or CD95; FasL, Fas ligand), both of which are linked to TNF receptor (TNFR) family receptors that are connected to extrinsic signals. Although the exact mechanism is not fully understood, DON has demonstrated an ability to hinder DNA and RNA synthesis^18^, ^28^. Moreover, You et al.^32^ raised the presumption that DON and T‐2 could achieve the “immune evasion” process to actively evade immune surveillance by immune cells. A recent study showed that T‐2 could initiate the “immune evasion” process by activating the signaling pathway involving the programed cell death protein‐1/programmed cell death‐ligand 1^45^. For DON, however, there is a dearth of related studies.

### FUMs

FUMs are a class of long‐chain amino polyalcohols that are primarily synthesized by species in the *Fusarium fujikuroi* species complex, especially in *Fusarium verticillioides* and *F. fujikuroi* (Figure 2). FUMs occur primarily in cereals (rice, wheat, barley, maize, rye, oat, and millet). Over 28 FUM homologs are known, and particular emphasis has been given to the B series, notably FUM B_1_ (FB_1_), FB_2_, and FB_3_ ^11^. FB_1_ is composed of a diester comprising propane‐1,2,3‐tricarboxylic acids (TCA) and 2‐amino‐12,16‐dime thyl‐3,5,10,14,15‐pentahydroxyleicosane, where hydroxyl groups at the C‐14 and C‐15 positions interact with the carboxyl groups of TCA to form an ester. Moreover, FB_2_ and FB_3_ can be regarded as the C‐5 and C‐10 dehydroxy analogs of FB_1_ ^19^ (Figure 3B). FUMs induce a range of deleterious effects on organisms, encompassing carcinogenicity, cytotoxicity, hepatotoxicity, immunotoxicity, nephrotoxicity, neurotoxicity, and reproductive toxicity^46^.

In *F. verticillioides*, the biosynthesis of FUMs necessitates a sophisticated gene cluster spanning 42 kb with 17 coregulated genes (Figure 4A). Except for *FUM20*, the FUM production profiles of FUM gene deletion or disruption mutants were ascertained using liquid chromatography–mass spectrometry (LC‐MS) analysis (Table 1). *FUM1*, which encodes a polyketide synthase (PKS), is the key gene in FUM biosynthesis. In the first step, the FUM1 protein (Fum1p) facilitates the condensation of 1 acetyl coenzyme A (acetyl‐CoA), 8 malonyl‐CoAs, and 2 *S*‐adenosyl methionines (SAMs) to generate a linear 18‐carbon‐long polyketide. In the second step, Fum8p catalyzes the condensation between the linear polyketide and alanine, resulting in a 3‐keto intermediate with a 20‐carbon chain. In the third step, Fum6p is responsible for catalyzing the hydroxylation process of the polyketide‐amino acid condensation product at C‐14 and C‐15. Then, the carbonyl group at C‐3 is reduced into an alcohol group by Fum13p. The addition of a hydroxyl group to the C‐10 carbon is catalyzed by Fum2p. The catalysis of esterification, leading to the hydroxylation at the C‐14 and C‐15 positions of the FUM backbone, is facilitated by Fum14p. In addition, the involvement of Fum7p, Fum10p, and Fum11p is also evident in the biosynthesis of the tricarballylate portion. The final step is the hydroxylation of the FUM backbone at C‐5 by Fum3p (Figure 4C). Fum21p, a transcription regulator with a Zn(II)2Cys6 DNA‐binding domain, can positively regulate the gene expression of *FUM1* and *FUM8* and is also required for FUM synthesis^21^.

FB_1_, the most typical FUM, has a structure similar to that of sphingolipids (SLs), and it has the capability to act as an inhibitor of ceramide synthase (CerS). Inhibition of CerS interferes with SL metabolism, causing the accumulation of free sphinganine (Sa) and sphinganine‐1‐phosphates (Sa‐1‐P) in cells, alterations in complex SLs, and reduced levels of ceramides^46^. FB_1_ and its generated toxic products (Sa, Sa‐1‐P) induce oxidative stress by exacerbating peroxide production (ROS, H_2_O_2_, lipid peroxide, and lipid oxidation end products) and inhibiting the activity of antioxidants (SOD, CAT, GSH‐PX, and GSH^46^; Figure 5B). FB_1_‐induced oxidative stress can induce JNK phosphorylation, activate P53 signaling, and upregulate the expression of proapoptotic factors (P53‐upregulated modulator of apoptosis [PUMA] and Caspase 3) to cause apoptosis^41^, ^47^. FB_1_‐induced EnR stress leads to apoptosis through the JNK/p53/PUMA/Caspase 3 pathway and autophagy through the IRE1/JNK pathway, which releases Beclin‐1 (BECN1) from the Bcl‐2‐BECN‐1 interaction to promote the conversion of microtubule‐associated protein 1 light chain 3 (LC3)‐I into LC3‐II^47^. In addition, FB_1_‐induced EnR stress can induce autophagy by the PERK pathway and the AMP‐dependent protein kinase (AMPK) pathway^47^, ^48^. FB_1_ exposure can also induce apoptosis by regulating the phosphatase and tensin homolog (PTEN)/PI3K/AKT signaling pathway via disruption in lipid raft formation^49^. Moreover, FB_1_ exerts an epigenetic influence on the PTEN/PI3K/AKT signaling pathway to enhance DNA damage by inhibiting checkpoint kinase 1 (CHK1) activity through phosphorylation of its Ser280 residue, thereby impeding the repair process for damaged DNA^50^. It has also been demonstrated that the apoptotic pathway of FB_1_ is linked to death receptor pathways, including the TNF pathway and the Fas pathway^46^. In the TNF pathway, the function of NF‐κB is complex. Gopee et al.^51^ suggested that FB_1_‐induced apoptosis involved the activation of Caspase 3 in pig kidney epithelial cells (LLC‐PK_1_), which was correlated with the suppression of NF‐κB. However, Chen et al.^52^ suggested that FB_1_ treatment resulted in the upregulation of both Caspase 3 and NF‐κB in pig kidney (PK‐15) cells.

### ZEA

ZEA, formerly known as F‐2 toxin, is a resorcylic acid lactone (Figure 3C) that is synthesized by some members of the *F. sambucinum* species complex, the *F. incarnatum‐equiseti* species complex, and the *F. fujikuroi* species complex (Figure 2), such as *F. graminearum, F. culmorum, F. equiseti*, and *F. verticillioides* ^11^. The toxicity of ZEA encompasses various dimensions, including alimentary canal toxicity, endocrine interference, carcinogenicity, genotoxicity, hepatotoxicity, immunotoxicity, and reproductive toxicity. ZEA predominantly contaminates grains, including maize, wheat, rice, barley, sorghum, soybean, oat, and their products^53^.

In *F. graminearum*, the ZEA biosynthesis gene cluster contains four genes: *PKS13*, *PKS4*, *ZEB1*, and *ZEB2* (Figure 4A). Disruption of *PKS13*, *PKS4*, or *ZEB2* can result in a permanent halt in ZEA production, and the *ZEB1* deletion mutant produces the ZEA derivative β‐zearalenol (β‐ZEL; Table 1). PKS4 can catalyze the synthesis of the hexaketide chain using one acetyl‐CoA and five malonyl‐CoA units. Then, the hexaketide is transferred to the nonreducing PKS13 to form nonaketide after completing three condensations. The nonaketide subsequently undergoes two successive intramolecular cyclization reactions, leading to the formation of an aromatic ring and a macrolide ring structure containing a lactone bond. Finally, β‐ZEL is transformed to ZEA by ZEB1, which facilitates the transformation of the hydroxyl group on the macrolide into the ketone group^23^ (Figure 4D).

ZEA and its metabolites have a three‐dimensional (3D) structural similarity to estradiol and can exert estrogen‐like effects. Estrogen regulates physiological processes via estrogen receptors (ERs), which are capable of initiating many signaling pathways. At low concentrations, ZEA usually induces the proliferation of cells through estrogen‐like effects and carcinogenic properties^54^. ZEA and its metabolites can occupy and activate ERs and then mediate the expression of estrogen‐responsive genes through the ERK signaling pathway^55^ (Figure 5C). The modulation of physiological estrogen responses, such as endocrine disruption and cell proliferation, is attributed to the expression of genes regulated by estrogen^56^. The DNA damage caused by ZEA might lead to mutations or chromosome abnormalities, which can disturb the progression of the cell cycle and cause cell proliferation or cell cancerization^57^, ^58^. Furthermore, ZEA downregulates the expression of tumor suppressor genes and upregulates the expression of oncogenes in TM3 cells, potentially promoting the conversion of normal cells into malignant cells^59^. Similarly, a change in oncogene expression might cause cell proliferation. Moreover, DNA damage caused by ZEA can also lead to cell cycle arrest. DNA damage causes the upregulated expression of ataxia‐telangiectasia mutated serine/threonine kinase (ATM) and ataxia telangiectasia and Rad3‐related protein (ATR), which activate CHK1 and CHK2, and cells begin to repair the damage. However, ZEA‐exposed cells undergo arrest in the G2/M phase, during which there are upregulated expression of cell division cycle 25 phosphatases (CDC25) A and CDC25C. These two proteins subsequently enhance the expression of cyclin B1 (CCNB1) and cyclin‐dependent kinase 1 (CDK1), thereby preventing exit from the G2/M phase of the cell cycle. The arrest of cell cycle progression triggers a halt in DNA replication and consequently inhibits cellular proliferation^60^, ^61^, ^62^. At high concentrations, ZEA causes mitochondrial dysfunction, EnR stress, apoptosis, and autophagy. ZEA reduces the protein expression of Nrf2 and HO‐1 to further induce oxidative stress and cause cell apoptosis via the p38, JNK, and ERK MAPK pathways^63^, ^64^, ^65^. Bai et al. found that ZEA can induce apoptosis by modulating EnR stress though the PERK and ATF6 signaling pathways in porcine trophectoderm cells^66^. In addition to the classical signaling pathways, ZEA induces apoptosis through the ERK/p53/Caspase 3 signaling pathway and the Caspase 12 signaling pathway activated by Ca^2+^ release from the EnR^67^, ^68^. ZEA‐induced EnR stress also causes autophagy through the PERK pathway^69^. Moreover, ZEA can cause apoptosis by activating death receptor pathways and inhibiting the PI3K/AKT pathway and can induce the expression of proinflammatory cytokines through the TNF/NF‐κB pathway and MAPK/NF‐κB pathway^70^, ^71^, ^72^, ^73^.

ZEA can be biotransformed in the liver and bacterial gut flora of mammals by hydroxysteroid dehydrogenases (HSD). There are 4 reductive metabolites of ZEA in the reductive phase‐I metabolism process: α‐ZEL, β‐ZEL, α‐zearalanol (α‐ZAL, also known as zeranol), and β‐ZAL (Figure 3C). 3α‐ and 3β‐HSD can catalyze the hydroxylation of ZEA, resulting in stereoisomeric compounds α‐ and β‐ZEL, respectively. Furthermore, with the saturation of a double bond, α‐ and β‐ZEL can be further reduced into α‐ and β‐ZAL, respectively (Figure 4E). α‐ZEL and α‐ZAL show higher xenoestrogenic effects than ZEA, while β‐ZEL and β‐ZAL are just the opposite. α‐ZAL has been extensively used as a growth enhancer to augment the fattening rates of cattle. Since 1985, the application of α‐ZAL has been banned in the European Union.^74^

## CC AND *FUSARIUM* MYCOTOXIN CONTAMINATION

**Figure 6:**
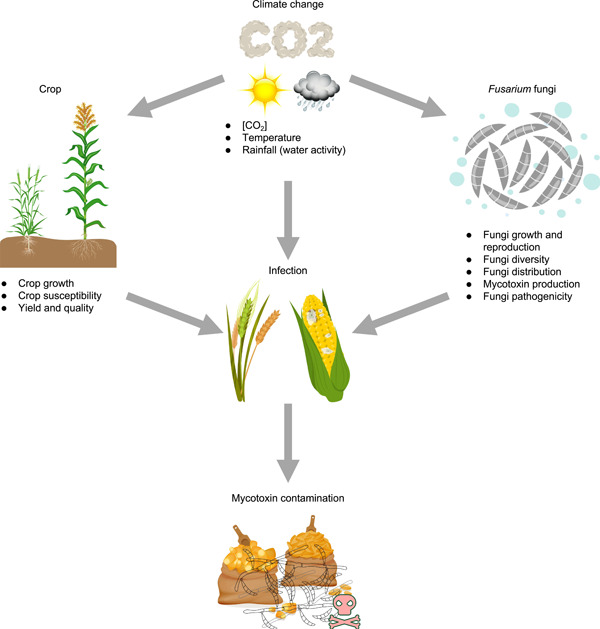
The impact of climate change on *Fusarium* mycotoxin contamination. Temperature, rainfall (or water activity), and carbon dioxide concentration ([CO_2_]) are the main known climate drivers affecting *Fusarium* mycotoxin contamination. Climate factors not only influence the growth and development of crops and *Fusarium* fungi but also affect the infection process of *Fusarium* fungi through changes in crop susceptibility, virulence of *Fusarium* fungi, and environmental factors at critical stages of infection.

On the basis of the Sixth Assessment Report of the Intergovernmental Panel on Climate Change, it is projected that global temperatures will continue to rise, while atmospheric concentrations of carbon dioxide ([CO_2_]) are anticipated to undergo a twofold or even threefold increase within the next 25–50 years^75^. Adverse CC can lead to more frequent occurrences of extreme weather events, such as heat waves, cold waves, heavy rainfall, and drought. CC is a global challenge and is forecasted to have significant impacts on food security by influencing crop growth, the occurrence of pests and diseases, and mycotoxin contamination. CC may lead to transformations in the spatial distribution and manifestation of mycotoxigenic fungi, thereby causing alterations in both the geographical distribution and the occurrence pattern of mycotoxins. Studies have shown that temperature, water activity (*a*_w_), [CO_2_], or a combination of the three variables can also impact the growth, proliferation, and production of mycotoxins from mycotoxigenic fungi^76^ (Figure 6). Although pathogenic *Fusarium* species are important producers of mycotoxins, there is no clear correlation, in general, between disease severity and mycotoxin contamination^77^. At Bottelare village, East Flanders province, Belgium, a positive correlation between FHB severity and DON content was observed during the growing seasons of 2001–2002 and 2002–2003; however, this relationship was not evident in the 2003–2004 season^78^.

### Temperature and rainfall

Although FUMs, DON, and ZEA have been detected all over the world, because of the different optimal production temperatures and *a*_w_ values of *Fusarium* fungi (Table 2), the contaminants have clear differences in geographical distribution. FUMs are the main *Fusarium* mycotoxin contaminants in southern Europe, the Americas, the Middle East, Africa, and south and southeast Asia. DON contamination occurs mostly in northern, central, and eastern Europe, northern and central America, South Africa, and East Asia. Compared to those of FUMs and DON, the occurrence rate of ZEA is lower and is only over 50% in East Asia and sub‐Saharan Africa^79^.

**Table 2:** The optimal temperature and *a*_w_ of *Fusarium* mycotoxin production.

	*Fusarium verticillioides*		*Fusarium proliferatum*		*Fusarium graminearum*		*Fusarium culmorum*	
Mycotoxin	Temperature (°C)	*a*_w_	Temperature (°C)	*a*_w_	Temperature (°C)	*a*_w_	Temperature (°C)	*a*_w_
FUMs	15–32	>0.940	13–25	>0.960				
DON					15–35	>0.980	20–30	>0.975
ZEA					25–30	>0.980		

The optimal temperature and *a*_w_ of FUMs and ZEA production were detected on *Fusarium*‐infected maize grain. The optimal temperature and *a*_w_ of DON production were detected on *Fusarium*‐infected wheat grain. Data were extracted from Sanchis and colleagues^80^, ^81^. *a*_w_, water activity; DON, deoxynivalenol.

Against the backdrop of global warming, regions currently characterized by cooler climates will witness the prevalence of toxigenic fungi that show optimal growth and mycotoxin production under higher‐temperature conditions. Conversely, areas already experiencing hot temperatures might observe a decrease in the occurrence of such fungi. With rising temperatures, lower crop yield and quality will occur in some regions that are currently considered warm. This might result in a reduction in total mycotoxin production due to the reduced crop quantity. However, due to the lower quality of crops, the mycotoxin content per unit weight of crops might increase^82^. Temperature is closely related to latitude and altitude. In most regions where maize is grown, FUM contamination tends to be higher in areas with lower latitudes and altitudes due to relatively warmer conditions compared to regions with higher latitudes or altitudes^83^. In the United States, the FUM risk is higher in Texas and the southeastern states than in the central states^84^. Shelby et al.^85^ discovered a significant inverse relationship between latitude and FUM concentration in the United States. A similar pattern can also be found in Asia, north of the Tropic of Cancer. In the low‐altitude maize production areas of central and south America, as well as southeast Asia, FUMs emerge as a significant risk factor. In Guatemala, a survey of maize samples gathered from fields between 2000 and 2003 showed that lowland maize exhibited significantly higher levels of FB_1_ than highland maize did^86^. In Europe, the risk of FUM contamination is higher in Italy, Spain, and southern France. In Africa, all maize‐producing areas are at risk for FUMs, with severity depending on altitude^84^. A survey in Uganda found that the FUM contamination of maize was widespread and that maize from high altitudes showed the most significantly elevated levels of FUM content^87^. DON contamination is also sensitive to temperature changes. In northwestern European countries, according to the prediction model, the flowering and full maturation of wheat will advance with the relative increase in temperature, and most regions will show a substantial rise in DON contamination in the 2040s^88^.

Although numerous studies have demonstrated that *Fusarium* mycotoxin production requires high *a*_w_ in vitro (Table 2)^80^, ^81^ , none have demonstrated monotonic relationships between rainfall and *Fusarium* mycotoxin contamination across different environments. In Ontario, Canada, a cool maize‐growing region, FUMs are only present in drought‐stressed fields^89^. In the United States, there is a significant negative correlation between June rainfall and FUM content at multiple locations^85^. Akello et al.^90^ studied the FUM content of cereals in Zimbabwe during 2015 and 2017 and found that FUM contamination was higher in wet years than in dry years. According to existing meteorological data in the Philippines, Salvacion et al.^91^ built a risk model of FUM contamination on corn using a fuzzy logic methodology. Due to the increased rainfall, they argued that a substantial proportion of the Philippines might be at a very high risk under prevailing circumstances, as well as under the anticipated CC scenarios for 2050. During the period 2012–2021, in both Serbia and Croatia, the highest mean contents of DON and ZEA in maize were observed in 2014, which might be related to the extreme precipitation during that year^92^. A 10‐year (2008–2017) global survey showed that maize harvested in central and southern Europe exhibited elevated levels of DON and ZEA concentrations in 2014, which corresponded to higher rainfall in July 2014. In the primary maize‐growing regions of China, DON and ZEA levels were relatively low in 2013, which might be related to the decrease in precipitation during August and September of that year^79^.

The period around flowering is considered to be the pivotal stage for *Fusarium* infection of cereals. Therefore, many studies have been dedicated to predicting the concentrations of *Fusarium* mycotoxins by the weather variables of the period around flowering, especially temperature and rainfall (Table 3). Campa et al.^93^ built a model to predict the FUM concentration of maize by using data gathered from Argentina and the Philippines. The model showed that weather was the major variable in total FUM concentration; the temperature and precipitation of the four periods around silking were determined to be the key factors in the FUM concentration. Hooker et al.^94^ identified a comprehensive set of weather variables and their temporal patterns for the prediction of DON contamination in mature wheat grain in Canada. They found that contamination showed a significant correlation with weather conditions during three pivotal periods around the heading stage. In the first period, 4–7 days before heading, DON generally decreased with the number of days with temperatures below 10°C and increased with the number of days experiencing rainfall exceeding 5 mm. In the second period, 3–6 days after heading, DON levels showed an upward trend in correlation with an increase in the number of days with rainfall exceeding 3 mm and a downward trend when exposed to temperatures exceeding 32°C. In the third period, 7–10 days after heading, DON increased with an increase in days with rainfall exceeding 3 mm. In Schleswig‐Holstein, northern Germany, Birr et al.^95^ found that there were significant relationships between the two weather variables (cumulative precipitation and average temperature during the period of wheat flowering) and the concentrations of ZEA in wheat grain at harvest. Based on this finding, they derived weather‐based forecasting models for predicting ZEA levels in wheat grain during the harvest stage for various *Fusarium*‐susceptible wheat cultivars. Joo et al.^96^ constructed a model to assess the influence of CC on ZEA contamination in rice grains cultivated in South Korea. The results indicated that increased temperature and relative humidity during the rice heading period and fluctuations in daily temperature throughout the harvest season can increase ZEA contamination in rice. The forecasts showed that ZEA contamination of rice could increase nationwide in both the 2030s and the 2050s, particularly in the western region of South Korea.

**Table 3:** The conditions for predicting the impact of CC on *Fusarium* mycotoxin contamination.

Mycotoxin	Crop	Critical period	Increased danger	Decreased danger	Ref.
FUMs	Maize	(1) 4 to 10 days before silking	RAIN >2 mm^a^	TMIN < 15 °C^b^, TMAX > 34 °C	[91]
		(2) From 4 days before silking to 2 days after silking	TMAX > 34°C		
		(3) 2 to 8 days after silking	TMAX > 34°C	RAIN >2 mm, TMIN < 15 °C	
		(4) 8 to 14 days after silking		RAIN >2 mm	
DON	Wheat	(1) 4 to 7 days before heading	RAIN >5 mm	TMIN < 10 °C	[92]
		(2) 3 to 6 days after heading	RAIN >3 mm	TMAX > 32 °C	
		(3) 7 to 10 days after heading	RAIN >3 mm		
ZEA	Wheat	Flowering period	The higher cumulative precipitation and average temperature		[93]
	Rice	(1) Flowering period	The higher average temperature and humidity		[94]
		(2) Harvest period	The higher daily temperature changes		

^a^ The risk of *Fusarium* mycotoxin contamination will increase with the number of days experiencing rainfall exceeding 2 mm. ^b^The risk of *Fusarium* mycotoxin contamination will decrease with the number of days when the minimum temperature is below 15 °C.

### [CO_2_] level

Although the impacts of elevated [CO_2_] on crops have been studied in depth^97^, there remains a scarcity of studies examining the responses of plant disease to increased [CO_2_]. Furthermore, research that specifically focuses on mycotoxins in plants is even more limited. Currently, the effects of [CO_2_] on *Fusarium* mycotoxin contamination have mainly been studied in laboratories; large‐scale field surveys have not yet been conducted. Elevated [CO_2_] can increase plant susceptibility to *Fusarium* species attacks. Rising [CO_2_] was found to increase *F. verticillioides* proliferation in maize with no change in FUM levels. This result indicates a decrease in the production of FUMs per unit of pathogen. Following *F. verticillioides* infection at elevated [CO_2_], the suppression of maize 13‐lipoxygenase and jasmonic acid production was correlated with a decrease in terpenoid phytoalexins and an increase in susceptibility to the pathogen, while a reduction in 9‐lipoxygenase, previously proposed to enhance mycotoxin production, was responsible for reduced FUMs per unit fungal biomass^98^. Further research showed that concurrent elevated [CO_2_] and drought stress significantly augmented the susceptibility of maize to *F. verticillioides* infection, consequently leading to an escalated contamination of FUMs. However, the negative impacts of drought on the accumulation of maize phytohormones and metabolites were not mitigated by elevated [CO_2_], and there was still no observed increase in FUMs per unit fungal biomass. Therefore, it is likely that the escalation in FUM contamination can be attributed to the greater *F. verticillioides* biomass^99^. *F. graminearum* infection produced similar results. Hay et al.^100^ found that elevated [CO_2_] can significantly increase *F. graminearum* biomass and DON accumulation in maize, but the DON per unit fungal biomass was unaffected. For wheat, it was a different story. In the *F. culmorum* single‐floret inoculation treatment, the concentration of DON was significantly increased under elevated [CO_2_]. This result suggests that the DON content is not directly related to the level of infection with *F. culmorum* ^101^. In addition, acclimatization to elevated [CO_2_] can impact the mycotoxin production of *Fusarium* fungi. A recent in vitro study demonstrated that, under elevated [CO_2_] conditions, *F. sporotrichioides* had a greater ability to produce T‐2 and HT‐2 after 10 subculture generations than in the initial subculture of the strain^102^.

## DETECTION OF *FUSARIUM* MYCOTOXINS

Typically, analysis methods for mycotoxins require three major steps: extraction, cleanup, and detection^103^. Mycotoxins can be detected using various techniques, mainly chromatographic methods, immunological methods, and biosensor technologies (Table 4). However, each approach has advantages and disadvantages. The selection of a particular method is contingent upon the specific detection requirements^20^.

**Table 4:** The detection methods of main *Fusarium* mycotoxins.

	Detection method					Time (min)				
Mycotoxin	Chromatography	Immunoassay	Biosensor	Matrix	Sample preparation method	Preparation	Assay	LOD (ppb)	Specificity	Ref.
FB_1_	LC‐MS/MS			Corn	SLE	92	21	8		[104]
				Corn	SLE	120	30	100		[105]
				Corn	SLE	100	15	3		[106]
				Peanut	SLE	120	30	5		[105]
				Pistachio	SLE	120	30	10		[105]
				Wheat	SLE	120	30	10		[105]
				Wheat	SLE	100	15	0.5		[106]
				Raisin	SLE	120	30	5		[105]
	HPLC‐MS/MS			Wheat flour	SLE	>65	30	12		[107]
				Wheat flour	SPE	>30		0.01		[108]
				Corn	SPE	>11	25	0.64		[109]
	HPLC‐FLD			Corn	SLE	>15		50		[110]
				Tortilla, masa, corn	SLE	>45		25		[111]
				Canned sweet corn, fresh sweet corn, corn grits, corn flour, cornflakes	SLE	180	25	29.2		[112]
				Cereal foods	IAC		20	14.6		[113]
	UPLC‐MS/MS			*Alpinia oxyphylla*	SLE	35	32	0.2		[114]
				Corn	QuEChERS	60	25	6.3		[115]
		ELISA		Corn	SLE	15	1110	8	224% and 73% CRs with FB_2_ and FB_3_	[110]
				Corn and corn related samples	SLE	50	960	1	5% CR with T‐2	[116]
				Corn	SPE		120	0.19	6.89% and 2.93% CRs with FB_2_ and FB_3_	[117]
				Corn	SLE	40	915	1.15	60.4% CR with FB_2_	[118]
				Corn, feedstuff, wheat	SLE	10	730	1.18	The negligible CRs with FB_2_, OTA, ZEA, DON, and AFB_1_	[119]
		LFI		Corn	SLE	15	10	25	No CRs with ZEA, DON, OTA, AFB_1,_ and FB_1_	[120]
				Corn	SLE	30	10	0.5	No CRs with AFM_1_, DON, FB_2_, T‐2, and FB_3_	[121]
				Feed	SLE	30	10	1.94	AFM_1_, DON, FB_2_, T‐2, and FB_3_ did not interfere with the detection of FB_1_	[121]
				Corn, wheat	SLE	15	5	20	AFB_1_, ZEA, and OTA did not interfere with the detection of FB_1_	[122]
				Chinese traditional medicine	SLE	25	5	5	No CRs with AFB_1_, ZEA, and OTA	[123]
		MIP‐ELISA		Corn	SLE		1445	1.9 × 10^−3^	The negligible CRs with FB_2_, AFB_1_, CIT, ZEA, and DON	[124]
			MIP‐EC biosensor	Corn	SLE	6		8.89 × 10^−6^	The negligible CRs with AFB_1_, CIT, DON, and ZEA	[125]
			MIP‐ECL biosensor	Milk, corn	LLE, SLE	30	15	3.5 × 10^−4^	The negligible ECL signals of OTA, OTB, DON, CS, LAC, DA, and NE	[126]
			MIP‐PEC biosensor	Milk, corn	LLE, SLE	30	20	4.7 × 10^−3^	The negligible photocurrents of OTA, OTB, DON, ZEA, PAT, Glu, and starch	[127]
			EC immunosensor	Corn	SLE	20	40	4.2	No response for DON	[128]
				Corn	IAC		180	0.002	The peak currents caused by ZEA, OTA, and DON showed a comparable pattern to that observed in the control sample	[129]
			EC aptasensor	Beer			10	2.6 × 10^−4^	The peak current of OTA, ZEA, and AFB_1_ was higher significantly than FB_1_	[130]
				Rice	SLE	45		8.7 × 10^−5^	The obvious differences of ECL signals between FB_1_ and AFB_1_, AFB_2_, DON, OTA, ZEA	[131]
			Colorimetric signal aptasensor	Corn, wheat	SLE	25	30	0.024	Effectively avoiding interferences of FB_2_, AFB_1_, DON, ZEA, and T‐2	[132]
			ECL aptasensor	Wheat	SLE			0.27	The obvious differences of ECL signal between FB_1_ and OTA, AFT, l‐cys, l‐Hcys	[133]
DON	GC‐MS			Wheat	SPE	90	24.2	3		[134]
	LC‐MS/MS			Corn	QuEChERS	13	44	739		[135]
				Corn	SLE	120	30	50		[105]
				Corn	SLE	100	15	8		[106]
				Peanut	SLE	120	30	75		[105]
				Pistachio	SLE	120	30	50		[105]
				Wheat	SLE	120	30	20		[105]
				Wheat	SLE	100	15	35		[106]
				Raisin	SLE	120	30	9		[105]
	UPLC‐MS/MS			*Alpinia oxyphylla*	SLE	35	32	6		[114]
				Corn	SPE	26	9	0.1		[136]
				Oat	SPE	26	9	0.12		[136]
				Corn	QuEChERS	60	25	3.2		[115]
	HPLC‐MS/MS			Wheat flour	SLE	>65	30	5.1		[107]
				Corn	SPE	>11	25	0.29		[109]
	HPLC‐FLD			Wheat	IAC			21.7		[137]
				Corn	IAC			14.08		[137]
	HPLC‐PDA			Cereal foods	IAC		30	15.5		[113]
		ELISA		Wheat	SLE	>15	45	0.62	4.7% CR with 3ADON	[138]
				Cereals and cereal products	SLE	20	790	4.9	5.7% CR with 3ADON	[139]
				Rice	SLE	20	300	0.94		[140]
				Rice, corn, flour, feed	SLE	45	835	0.2	80.34%, 2.17%, and 2.74% CRs with 3ADON, 15ADON, and FUS‐X	[141]
		LFI		Corn, wheat	SLE	7	5	100	No CRs for multianalysis of DON and ZEA	[142]
				Corn, wheat	SLE	15	5	5	AFB_1_, ZEA, and OTA did not interfere with the detection of DON	[122]
				Corn, wheat	SLE	>8	10	50	400%, 1.6%, and 4.3% CRs with 15ADON, 3ADON, and NIV	[143]
				Chinese traditional medicine	SLE	25	5	5	NO CRs with AFB1, ZEA, and OTA	[123]
				Rice, corn	SLE	45	15	12.5	80.34%, 2.17%, and 2.74% CRs with 3ADON,15ADON, and FUS‐X	[141]
			SPR immunosensor	Corn, wheat	SLE	45	20	3.26	16.2% CR with 15ADON	[144]
			EC immunosensor	Wheat	SLE	30	13	342.4	221% CR with 3ADON	[145]
			MIP‐EC biosensor	Corn	SLE	25	15	0.3	Compared to OTA, FB_1_, FB_2,_ NIV, and ZEA, MIP sensor showed higher recognition selectivity toward DON	[146]
				Wheat flour	SLE	40	6.5	0.021	The ΔI after incubation in DON is exhibited higher than that in ascorbic acid, Cu^2+^, Glu, glutamic acid, OTA, K^+^, Na^+^, Mg^2+^, sucrose, and ZEA	[147]
			MIP‐SPR biosensor	Standard substance				1	19% and 44% selectivity efficiencies with 3ADON and 15ADON	[148]
			SERS aptasensor	Corn flour, peanut oil, pure milk	LLE, SLE	>40	40	3.2 × 10^−5^	The obvious differences of SERS signal between DON and AFB_1_, OTA, FB_1_, T‐2, and ZEA	[149]
				Wheat flour	SLE	15	40	0.06	The obvious differences of SERS signal between DON and ZEA, OTA, AFB_1_, T‐2, FB_1_	[150]
			FL aptasensor	Corn flour	SLE	30	45	1.87	The restored FL intensity of DON showed a significantly higher value compared to AFB_1_, OTA, T‐2, and ZEA	[151]
				Wheat flour	SLE	15	40	0.08	The obvious differences of FL signals between DON and ZEA, OTA, AFB_1_, T‐2, FB_1_	[150]
			EC aptasensor	Corn flour	SLE	45	90	6.9 × 10^−6^	The obvious differences of current between DON and ZEA, T‐2, AFB_1_, FB_1_	[152]
ZEA	LC‐MS/MS			Peanut	SLE	120	30	5		[105]
				Pistachio	SLE	120	30	10		[105]
				Corn silage	QuEChERS	13	44	9		[135]
				Wheat	SLE	120	30	5		[105]
				Wheat	SLE	100	15	1		[106]
				Corn	SLE	120	30	10		[105]
				Corn	SLE	100	15	0.5		[106]
				Raisin	SLE	120	30	2		[105]
	LC‐FLD			Corn	ASE	13	15	6		[153]
				Wheat	ASE	13	15	6		[153]
				Rice	ASE	13	15	5		[153]
				Barley	ASE	13	15	3		[153]
	UPLC‐MS/MS			*Alpinia oxyphylla*	SLE	35	32	0.3		[114]
				Corn	QuEChERS	60	25	2.5		[115]
	HPLC‐MS/MS			Wheat flour	SLE	>65	30	1.6		[107]
				Wheat	SPE	90	24.2	2		[134]
				Wheat flour	QuEChERS	65.5	17	17.9		[154]
				Corn	SPE	>11	25	0.22		[109]
	HPLC‐FLD			Wheat	IAC			1.12		[137]
				Wheat, corn flakes, bread	SLE	23	20	2		[155]
				Corn	IAC			1.06		[137]
				Rice, wheat, oat, barley, corn	IAC			0.5	No interference from foreign peaks was observed at the retention times of AFB_1_, AFB_2_, AFG_1_, AFG_2_, OTA, and ZEA for the analytes	[156]
		ELISA		Corn, corn noodles, corn cookies	SLE	10	860	0.1	4.1%, 189.1%, and 43.9% CRs with α‐ZAL, β‐ZAL, and β‐ZEL	[157]
				Corn	SLE	>8	195	0.13	The negligible CRs with AFB_1_, DON, OTA, and T‐2	[158]
				Rice, barley, corn	SLE	>30	140	0.15	121.5%, 65.3%, 21.5%, and 18.9% CRs with α‐ZAL, β‐ZAL, α‐ZEL, and β‐ZEL	[159]
				Soybean meal, silage, sorghum, corn, distillers dried grains with soluble, total mixed ration	SLE	45	300	0.06	The CRs of less than 11% and less than 1% with zearalanone and ZAL	[160]
		LFI		Corn	SLE	30	11	3.6	The negligible CR with CIT, OTA, DON, FB_1,_ and AFB_1_	[161]
				Corn, wheat	SLE	7	5	6	No CRs for multianalysis of DON and ZEA	[142]
				Soybean meal, silage, sorghum, corn, distillers dried grains with soluble, total mixed ration	SLE	45	5	10	The CRs of less than 11% and less than 1% with zearalanone and ZAL	[160]
			SPR immunosensor	Wheat	SLE	45	20	7.07	15.3% and 11.5% CRs with α‐ZEL and β‐ZEL	[144]
			OWLS immunosensor	Corn	SLE	20		2 × 10^−6^	25.2%,12.8%, and 2.7% CRs with α‐ZEL, α‐ZAL, and β‐ZAL	[162]
			EC immunosensor	Standard substance			30	1.9 × 10^−3^	Less than 2.4% CRs with both DON and T‐2.	[163]
			MIP‐SPR biosensor	Corn			40	0.3	15%, 21%, 25%, and 27% selectivity efficiencies with α‐ZEL, β‐ZEL, α‐ZAL, zearalanone and α‐ZAL	[164]
			MIP‐ FL biosensor	Corn				5	35%, 3%, and 4% CRs with ZOL, OTA, and AFB_1_.	[165]
			MIP‐ EC biosensor	Corn	SLE	5	15	0.2	10%, 9%, 7%, 10%, and 14% CRs with NIV, OTA, FB_1_, FB_2,_ and DON.	[166]
			EC aptasensor	Beers				1.7 × 10^−4^	No obvious change of current with AFT, α‐ZAL, β‐ZAL, β‐ZEL, and OTA	[167]
			SERS aptasensor	Corn	SLE	20	210	6.4 × 10^−3^	The negligible Raman signal intensities with AFB_1_, OTA, DON, and FB_1_.	[168]
			FL aptasensor	Corn	SLE	30	150	0.126	The negligible fluorescent‐signal changes with α‐ZEL, β‐ZEL, ZEA‐4‐G, ZEA‐4‐S, AFB_1_, AFB_2_, OTA, FB_1_, and FB_2_	[169]
				Beer			150	0.007	The negligible fluorescent‐signal changes with α‐ZEL, β‐ZEL, ZEA‐4‐G, ZEA‐4‐S, AFB_1_, AFB_2_, OTA, FB_1_, and FB_2_	[169]

3ADON, 3‐acetyldeoxynivalenol; 15ADON; 15‐acetyldeoxynivalenol; AFB: aflatoxin B; AFG, Aflatoxin G; AFT, aflatoxin; ASE, accelerated solvent extraction; CR, cross‐reactivity; CS, casein; DA, dopamine; EC:electrochemical; ECL, electrochemiluminescence; ELISA: enzyme‐linked immunosorbent assay; FL: fluorescence; FLD, fluorescence detection; GC, gas chromatography; HPLC, high‐performance liquid chromatography; FUS‐X: fusarenon X; Glu, glucose; IAC: immunoaffinity column; L‐cys, L‐cystein; L‐Hcys, L‐homocysteine; LAC, lactose; LC, liquid chromatography; LFI: lateral flow immunoassay; LLE: liquid–liquid extraction; MIP: molecularly imprinted polymer; MS, mass spectrometers; MS/MS, tandem mass spectrometry; NE, norepinephrine; OTA, ochratoxin A; OTB, ochratoxin B; OWLS, optical waveguide light‐mode spectroscopy; PAT, patulin; PDA,photodiode array; PEC, photoelectrochemical; QuEChERS: quick, easy, cheap, effective, rugged, and safe; SERS: surface‐enhanced Raman spectroscopy; SLE: solid–liquid extraction; SPE, solid phase extraction; SPM, sample preparation methods; SPR: surface plasmon resonance; UPLC, ultra‐performance liquid chromatography; ZEA‐4‐S, zearalenone‐4‐sulfate.

### Extraction and precleaning methods

Primary extraction is essential for the determination of mycotoxins in various sample types. The cleanup step can eliminate interference from the extract and concentrate the analyte, and it is essential for the analysis of mycotoxins, especially at trace levels^102^. Currently, common approaches include solid phase extraction (SPE), multifunctional cleanup columns, liquid–liquid extraction (LLE), solid–liquid extraction (SLE), and immunoaffinity column (IAC)^170^. The selection of a method for mycotoxin extraction depends on the types of analytes. However, some methods can incur high costs, intricate procedures, and/or substantial time and solvent consumption. To minimize the sample treatment but prevent exposure to matrix effects, the “quick, easy, cheap, effective, rugged, and safe” (QuEChERS) sample preparation approach is a viable alternative. The QuEChERS method has been used for the extraction of mycotoxins from food samples, including dried fruits and cereals, as well as liquid samples, such as wine and beer^171^.

### Chromatographic methods

There are many kinds of chromatographic analytical methods for mycotoxin analysis, such as thin‐layer chromatography (TLC), high‐performance TLC (HPTLC), gas chromatography (GC), high‐performance liquid chromatography (HPLC), and ultra‐performance liquid chromatography (UPLC). HPLC has emerged as the most widespread technique for mycotoxin analysis. By coupling to detectors, such as mass spectrometry (MS), ultraviolet (UV) detectors, visible detectors, and fluorescence (FL) detectors (FLDs), the compounds separated by chromatography can be further identified^20^. Currently, LC‒MS and liquid chromatography FL detection (LC‒FLD) are widely recognized as the standard methods for detecting *Fusarium* mycotoxins. There is no doubt that FLD is the most sensitive among all LC detectors. The sensitivity, precision, and accuracy of LC‒MS may vary depending on the mycotoxins, matrix, ionization technique, and sensitivity of the process. Due to ion suppression and matrix effects, LC‒MS often causes undesirable results for the quantitative measurement of mycotoxins. Tandem mass spectrometry (MS/MS) is the preferred detection method over FLD due to its ability to identify a wide range of both fluorescent and nonfluorescent mycotoxins, making it a cost‐effective choice^103^. With the ongoing development of technology, high‐throughput determination methods of single or multiple matrices using LC‒MS/MS have been reported. Steiner et al.^172^ developed a pioneering multiclass quantitative method for the analysis of over 1200 biotoxins, pesticides, and veterinary drugs in complex feeds by LC–MS/MS.

In the past decade, liquid chromatography coupled with high‐resolution mass spectrometry (LC–HRMS) has turned from a research‐only technique into a costly tool for routine testing and high‐throughput food analysis in laboratories. Although these methods were initially developed for pesticide detection, mycotoxins are now the primary focus of LC–HRMS method development^173^. High resolution, in combination with the fast generation of product ion spectra, has the potential to minimize indistinct outcomes and streamline peak detection, but there remains a disparity between LC–HRMS and LC–MS/MS regarding the limits of detection (LOD) and of quantitation for most analytes. To address the gap for most analytes and enhance the applicability of mycotoxin trace analysis, various strategies for analyte enrichment, including SPE, have been incorporated into experimental protocols. However, these additional procedures invariably lead to a substantial augmentation in manual laboratory tasks and expenses, thereby diminishing the potential advantages of HRMS systems^173^, ^174^. Mateus et al.^175^ developed a UHPLC–HRMS multianalyte method for pistachio nuts. They evaluated different approaches to dispersive SPE for high‐lipid matrices; eventually, two procedures were validated. One involved the addition of enhanced matrix removal‐lipid for the detection of FUMs, and the other used a zirconium‐based material to achieve a slightly heightened sensitivity in analyzing G‐type AFs without including FUMs.

HPLC, UHPLC, and GC coupled with non‐MS detectors are also reference methods for *Fusarium* mycotoxin analysis. The current trend for the chromatographic analysis of *Fusarium* mycotoxins with non‐MS detection involves the advancement of multitoxin analysis with FLD, UV detection, photodiode array (PDA) detection, and so forth. Pi et al.^176^ developed a novel method for the simultaneous determination of nine mycotoxins based on ultrasonic‐assisted aqueous two‐phase extraction coupled with solidifying organic drop‐dispersible liquid–liquid microextraction by HPLC with a diode array detector and FLD in series. The methodology effectively identified various mycotoxins in multiple foods. Furthermore, it can be utilized to effectively perform regular and extensive analyses of numerous mycotoxins within various samples. Lee et al.^177^ built a simple and reliable HPLC‐UV method for the simultaneous determination of DON, NIV, DON‐3G, and NIV‐3G. This method involves a straightforward sample extraction with IAC purification and was successfully applied to analyze 31 different baby formulas and Korean rice wines available on the Korean market.

### Immunological methods

Immunological detection methods rely on the antibody–antigen (Ab–Ag) binding relationship and vary from enzyme‐linked immunosorbent assay (ELISA) and lateral flow immunoassay (LFI) to advanced immunosensors. ELISA is a technique utilized to detect the presence and quantity of Ag binding in biological samples based on the principle of Ag–Ab interactions. In recent decades, numerous ELISA kits have been effectively commercialized for the detection of *Fusarium* mycotoxins. ELISA tests are portable, simple, fast, and do not require expensive analytical equipment. This continues to make ELISA tests popular. Nevertheless, ELISAs often show limited precision at low concentrations, and structurally similar mycotoxins or matrices can impede conjugate and Ab binding, causing errors in quantifiable mycotoxin ELISA measurements^103^.

LFI is a simple one‐step immunochromatographic paper assay that does not require complex instruments. It can be classified into two modes, competitive and sandwich; typically, LFI for mycotoxin detection adopts the competitive type. The basic LFI equipment consists of sample coating pads, conjugate‐release pads, absorbent pads, and membranes (also called detection pads)^178^. For the user, LFI is strikingly simple: after simply adding the sample onto a single paper lateral flow strip and a short incubation time, the qualitative or semiquantitative result of the test is revealed by the appearance of a test line, and quantification can then be conducted using an optical reader^179^. The exceptional advantages of LFI in terms of convenience, affordability, and rapidity make it particularly suitable for on‐site monitoring and rapid testing for *Fusarium* mycotoxin contamination in foods. In recent years, the advancement of novel nanomaterials has broadened the types of labels available for LFI of *Fusarium* mycotoxins. Colored nanoparticle (NP)‐based LFI is simpler and more convenient, and it shows significant potential for on‐site detection^180^. Due to the intricate nature of mycotoxin co‐occurrence, there is a growing need for simultaneous detection of multiple mycotoxins. To date, several multiplex LFIs for mycotoxins with excellent performance have been effectively developed. For example, Liu et al.^181^ devised an innovative LFI integrated with gold nanoparticles (AuNPs) and time‐resolved FL microspheres. Their LFI is a smartphone‐based quantitative dual detection for multiplex mycotoxins in cereals, such as aflatoxin B_1_ (AFB_1_), ZEA, DON, T‐2, and FB_1_.

### Biosensors

Biosensors are bioanalytical devices that incorporate biological recognition elements to bind target molecules and a signal transducer for converting the biorecognition event into a quantifiable signal. Biorecognition elements, including Abs, Ags, nucleic acids, and enzymes, are used for the identification and detection of target analytes. Based on a variety of bioinspired recognition elements, biosensors can be classified into immunosensors, aptasensors, and molecularly imprinted polymer (MIP)‐based sensors^179^. According to the principle of signal transduction, biosensors can be divided into electrochemical (EC) biosensors, optical biosensors, mass‐sensitive biosensors, thermal biosensors, and so on^182^. Compared to the other analytical methods mentioned above, biosensors offer a much simpler and more efficient means of dynamically monitoring reaction changes in real‐time with digital outputs. Biosensors not only reduce detection time but also enhance sensitivity, simplicity, robustness, and reusability, enabling the development of cost‐effective, high‐throughput screening methods for mycotoxins^179^.

Immunosensors, as novel and widely utilized analytical instruments, use Ab as the recognition element and a transducer to convert the Ag–Ab binding event into a quantifiable physical signal. Due to the superior specificity of the Ag–Ab immunoreaction, immunosensors show superior selectivity and sensitivity^183^. Various immunosensors have been developed based on the different mechanisms of signal variations, including FL, colorimetric, chemiluminescence, electrochemiluminescence, surface plasmon resonance (SPR), surface‐enhanced Raman spectroscopy (SERS), and EC immunosensors. By combining the exceptional specificity of Abs and the remarkable sensitivity of FL detection, FL immunosensors have recently emerged as highly favored contenders for mycotoxin detection^183^. With advancements and breakthroughs in nanotechnology, a diverse array of nanomaterial semiconductors, including semiconductor quantum dots (QDs), QD nanobeads (QBs), carbon dots (CDs), fluorescent metallic NPs, and upconversion nanoparticles (UCNPs), with unique photostability, bright FL, and good biocompatibility have garnered immense attention regarding their use in the construction of FL immunosensors^184^. Yang et al.^185^ devised a novel FL immunosensor detection platform that integrates multicolor UCNP barcoding technology with smartphone‐based portable devices for simultaneous analysis of multiple mycotoxins (AFB_1_, ochratoxin A [OTA], and ZEA). The quantitative detection platform demonstrated feasibility and reliability, with a LOD of 1 ng that surpassed the values obtained from standard assays. SERS is a surface‐sensitive vibrational spectroscopy technique for the detection and characterization of analytes that are adsorbed on or close to the surface of plasmonic nanostructures. SERS integrates the advantages of the molecular specificity of Raman spectroscopy and the optical sensitivity of plasmonic nanostructures to boost the Raman signal, greatly extending the role and application field of standard Raman spectroscopy^183^. SERS significantly amplifies the Raman signal, thereby expanding the scope and applicability of conventional Raman spectroscopy in a profound manner. SERS immunosensors, which integrate the SERS labeling technique with Ag–Ab specific interactions, have emerged as novel immunosensing devices for mycotoxins. Li et al.^186^ developed a SERS immunosensor to simultaneously detect AFB_1_, ZEA, and OTA by using AuNPs labeled with 5,5‐dithiobis(succinimidyl‐2‐nitrobenzoate) as a Raman reporter. The SERS immunosensor determination method demonstrated results that are consistent with conventional instrumental analysis.

Aptamers are novel recognition elements with exceptional affinity and specificity that have the potential to serve as recognition molecules in aptasensors (aptamer‐based biosensors) for the efficient and swift identification of various targets^187^. Aptamers can be divided into two categories, DNA/RNA‐based aptamers and peptide aptamers. Currently, aptamers for the detection of mycotoxins are a class of single‐stranded DNAs or RNAs that undergo screening through the systematic evolution of ligands by exponential enrichment, which can selectively bind to different ligands through noncovalent bonds^188^. The functional similarity of aptamers to Abs makes them widely applicable in the field of biosensors. Compared to Abs, aptamers are low cost, have a longer shelf life and good stability, even at elevated temperatures, and are easy to modify and synthesize^189^. As with immunosensors, according to the signal variation mechanism, aptasensors can also be classified as FL aptasensors, EC aptasensors, SPR aptasensors, SERS aptasensors, CEL aptasensors, and so on. EC aptasensors use electrodes as sensing units and electrochemical workstations as signal transformation systems^190^. Aptamers are typically immobilized on the surface of the electrode; the specific molecular interactions between molecules on the electrode surface result in the conversion of target binding into electrical signals. These electrical signals, such as current, resistance, potential, or capacitance, are transmitted to a computer for quantitative or qualitative analysis of the target^187^. Zhang et al.^191^ devised an electrochemical aptasensor‐based target‐induced strand displacement strategy to achieve highly sensitive detection of T‐2. The aptasensor was highly specific, stable, and suitable for T‐2 detection in real samples. SPR is a phenomenon in which the electrons in a metal surface layer are excited by photons of incident light with a certain angle of incidence and then propagate parallel to the metal surface. With a constant light source wavelength and a thin metal surface, the angle that triggers SPR depends on the refractive index of the material near the metal surface. The affinity binding interaction on the surface of thin metal films can cause a small change in the reflective index of the sensing medium, which can hinder the occurrence of SPR. SPR aptasensors can detect those changes on the optical transducer surface, and optical transduction can directly convert the molecular binding event into a physically measurable signal, which is proportional to the concentration of analyte molecules^192^. As a label‐free analytical strategy, SPR aptasensors have the capability to detect multiple mycotoxins simultaneously with real‐time monitoring, high sensitivity, good specificity, minimal sample preparation requirements, and high‐throughputdetection^192^. Wei et al.^144^ effectively and simultaneously detected OTA, DON, AFB_1_, and ZEA in wheat and corn using SPR aptasensors that demonstrated high sensitivity, good linearity, and specificity.

MIPs, also referred to as “plastic antibodies,” are bespoke synthetic receptors that use the molecular imprinting technique. Following the general molecular imprinting method, imprint molecules (templates) are added along with functional monomers and cross‐linkers, which are polymerized under appropriate conditions. After copolymerization, the templates are extracted, leaving the 3D matrix with cavities that possess a matching shape, size, and chemical functionality with the template. The resulting cavities possess the ability to selectively reassociate with the template upon subsequent exposure and become synthetic receptors^174^, ^193^. The interactions between the polymers and the templates are similar to those between Abs and Ags. MIPs provide a cost‐effective and easily prepared approach for targeted template recognition with outstanding biocompatibility, repeatability, and broad utility. MIPs are particularly robust in extreme environments, such as elevated pressures, fluctuations in pH levels, and exceptionally high or low temperatures. By integrating MIPs with various sensing reporter systems (electrical, EC, optical methods, etc.), a valuable device can be developed for monitoring or screening purposes^194^. Utilizing a blend of MIP membranes (ZEA‐selective urethane acrylate MIP membranes) and the Spotxel® Reader smartphone application (Sycasys Software GmbH), a miniature sensor was used to analyze the natural FL of ZEA in cereals in the field^195^. NPs show a high level of sensitivity, yet their selectivity is limited. Therefore, the combination of MIPs and NPs can result in MIP composites with high sensitivity and selectivity. To date, several NPs, including QDs, UCNPs, carbon NPs (CNPs), AuNPs, magnetic NPs, and metal‐organic frameworks, have played important roles in mycotoxin analysis with MIPs^193^. The molecularly imprinted polymer nanoparticle‐based assay (MINA) was developed for the determination of FB_1_—the molecularly imprinted nanoparticles replace the primary Ab used in a competitive ELISA. The MINA showed a high level of specificity and did not show any cross‐reactivity with other mycotoxins, including AFB_1_, CIT, DON, ZEA, and FB_2_. The results of the MINA agreed with those obtained using traditional ELISA and HPLC methods^196^. Calahorra‐Rio et al.^197^ developed a new molecularly imprinted magnetic nanobead that can specifically extract ZEA from river and tap water for further analysis using HPLC–FLD. Their findings indicate that the magnetic nanobead showed exceptionally accurate and consistent ZEA detection in real liquid samples.

## THE MANAGEMENT STRATEGY FOR *FUSARIUM* MYCOTOXIN CONTAMINATION

**Figure 7:**
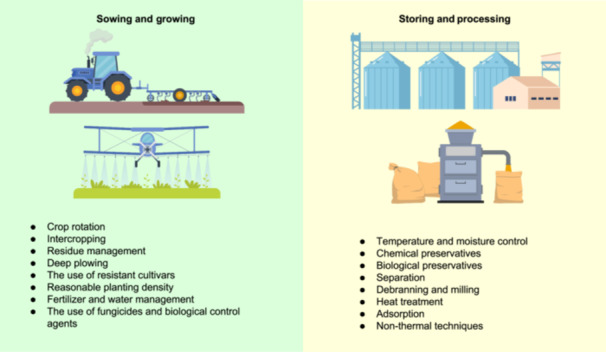
Proper practices to minimize *Fusarium* mycotoxin contamination. There are a series of management strategies that can be utilized to control *Fusarium* mycotoxins during the preharvest (sowing and growing) and postharvest stages (storing and processing). During the preharvest stage, these strategies place emphasis on preventing infection by *Fusarium* fungi. During the postharvest stage, management strategies are more focused on controlling the production of new mycotoxins and the detoxification and removal of existing mycotoxins.

*Fusarium* mycotoxins can be produced by several fungi in several stages of several crops, so a single management strategy cannot fully control *Fusarium* mycotoxin contamination. However, a series of practices, including agronomic, chemical, physical, and biological methods, can be implemented to avoid the spread of mycotoxins and to minimize their frequency in food products during preharvest and postharvest stages (Figure 7).

### Agronomic method

*Fusarium* fungi can survive in plant residues and on wild grasses, existing as mycelia, conidia, or perithecia. Therefore, residue management, tilling, and deep plowing can reduce the primary *Fusarium* inoculum that causes infections and the presence of mycotoxins^12^. Reduced or no tillage practices can contribute to increased DON contamination of wheat and maize^198^. Proper crop rotation can substantially mitigate *Fusarium* disease and mycotoxin contamination. An unfavorable practice in crop rotation is the consecutive cultivation of cereal plants^12^. Qiu et al.^199^ collected 90 wheat samples in China during 2013 and 2014, revealing that DON contamination was more prevalent in the rice–wheat rotation, while ZEA accumulation was found to be higher in the maize–wheat rotation. Crop rotation with noncereals can be a more favorable method for limiting *Fusarium* mycotoxin contamination; legumes, brassicas, and root crops might be better forecrops^12^. Moreover, the implementation of intercropping is a good method for controlling *Fusarium* mycotoxin contamination. Drakopoulos et al.^200^ found that the use of white mustard or Indian mustard as an intercrop with maize can reduce DON in winter wheat compared to maize grown as a sole crop in maize–wheat rotation fields under reduced tillage. The cultivation system, conventional or organic, also affects *Fusarium* mycotoxin contamination of cereals. Bernhoft et al.^201^ conducted a comprehensive analysis of available published studies (1991–2017) of controlled field experiments, comparing DON, ZEA, and T‐2/HT‐2 in grains from organic and conventional cultivation systems. Summary of the results revealed that organic production showed lower mycotoxin levels in 24 cases, no significant difference in 16 cases, and higher levels in only two cases.

### Host resistance

The most economical and sustainable approach to controlling *Fusarium* disease and mycotoxin contamination is through the cultivation of varieties that show resistance or partial resistance to *Fusarium* species, whether because of traditional breeding or through use of transgenic technology. Taking the case of FHB in wheat, there are five types of resistance: Type I (resistance to initial infection), Type II (resistance to disease spread within infected heads), Type III (resistance to mycotoxin accumulation), Type IV (resistance to kernel damage), and Type V (tolerance)^202^. These FHB resistance traits of wheat are governed by multiple quantitative trait loci (QTLs). Over 250 QTLs for FHB have been found on all 21 chromosomes in various wheat genotypes^203^. *Fhb1* on chromosome 3BS is the most important QTL. The Chinese wheat cultivar Sumai 3, known for carrying the *Fhb1* gene, is widely acknowledged as the superior source of FHB resistance and has been extensively utilized as a parent in numerous breeding programs^204^. Transgenic germplasms can offer novel supplementary resources for FHB management. Wheat resistance to *F. graminearum* could be significantly enhanced by overexpression of genes that encode defense signaling pathway‐related proteins, cell wall‐degrading enzyme inhibitors, and detoxification proteins^22^. McLaughlin et al.^205^ showed that the overexpression of *AtLTP4.4*, a nonspecific lipid transfer protein‐encoding gene of *Arabidopsis thaliana*, in transgenic wheat, can significantly reduce *F. graminearum* infection and DON contamination in the field. Based on RNA silencing, plants naturally have a defense system to stave off viral invasion. This feature has been harnessed to advance host‐induced gene silencing (HIGS) technology to control other plant pathogens by silencing pathogen genes in plants during infection. HIGS is also an effective control strategy against FHB in wheat. Recently, many researchers have successfully silenced important genes encoding cytochrome P450, chitin synthase, protein kinases, and others in *F. graminearum*, *Fusarium oxysporum*, *F. culmorum*, and *F. verticillioides*, resulting in reduced mycotoxin production^206^.

### Chemical method

Currently, due to the absence of efficacious disease‐resistant cultivars, the utilization of chemical fungicides remains the primary strategy for *Fusarium* disease management. There are several common types of fungicides for controlling *Fusarium* diseases, including benzimidazoles (benomyl, carbendazim, thiophanate‐methyl, and thiabendazole), triazoles (triadimefon, tebuconazole, diniconazole, and propiconazole), and strobilurins (azoxystrobin and pyraclostrobin). Strobilurin treatment can induce DON formation, so strobilurins are unlikely to be effective^207^. Triazoles, a group of demethylation inhibitor fungicides, are the gold standard for controlling FHB in wheat worldwide, while carbendazim is the most widely used fungicide in China for this purpose^207^, ^208^. In addition, phenamacril, a myosin I inhibitor and cyanoacrylate fungicide, is an efficacious and highly species‐specific fungicide for FHB in China. Phenamacril is not only capable of inhibiting the mycelial growth of a few *Fusarium* species, including *F. graminearum*, *Fusarium asiaticum*, *F. verticillioides*, and *F. oxysporum*, but also significantly disrupts DON‐toxisome formation to hinder DON biosynthesis^209^. Although spraying fungicides is a very effective method, the use of chemical fungicides comes with its own set of challenges. In addition to the well‐known environmental problems caused by fungicides, fungicide‐resistant population of *Fusarium* species are also an important concern. Due to prolonged and intensive use of fungicides, the benzimidazole‐ and triazole‐resistant strains of *F. graminearum* have become feared throughout world agriculture. There was a high frequency of carbendazim‐resistant *F. graminearum* in China. In response, the local government of Jiangsu Province, where large carbendazim‐resistant *F. graminearum* population had appeared, even proposed a fallow subsidy policy^210^. Regarding triazoles, tebuconazole‐resistant *F. graminearum* strains have been found in Argentina, China, Germany, and the United States^207^. Therefore, the development of new fungicides for the management of *Fusarium* diseases and mycotoxin contamination is urgently needed.

Chemical preservatives can be added to stored grain, particularly in the case of damp grains intended for animal feed. Common chemical preservatives include mixtures of aliphatic acid salts and antioxidants. The former has demonstrated efficacy in controlling FB_1_ production of *F. proliferatum* on irradiated maize kernels^211^. The two antioxidants, butylated hydroxyanisol and propyl paraben, were shown to reduce both the growth and FUM production of *F. verticillioides* and *F. proliferatum* in culture media and maize grain^212^. Recently, considering the adverse effects of synthetic preservatives, there has been a growing trend toward utilizing plant essential oils (EOs) as viable alternatives for mycotoxin management.

### Biological control method

Biological control is an eco‐friendly approach that uses living organisms or their derivatives to control pests. The useful biological control agents (BCAs) for *Fusarium* diseases and mycotoxin contamination include beneficial bacteria, fungi, actinomycetes, and mycoviruses. BCAs can protect crops against pathogenic *Fusarium* fungi before harvest, thereby effectively mitigating the risk of mycotoxin contamination in the food chain. The mechanisms of BCAs encompass competition for space and nutrients, mycoparasitism, synthesis of antifungal metabolites, cross‐protection, promotion of plant growth, and induction of host plant resistance^213^.

Among the bacterial BCAs, two genera that secrete antifungal compounds, *Bacillus* (such as *Bacillus velezensis*, *Bacillus megaterium*, and *Bacillus subtilis*) and *Pseudomonas* (including *Pseudomonas aeruginosa*, *Pseudomonas frederiksbergensis*, *Pseudomonas fluorescens*, and *Pseudomonas simiae*), are the most widely used and can effectively reduce *Fusarium* infection and mycotoxin contamination under field conditions^214^. *Bacillus* contain three important antifungal lipopeptides that inhibit *Fusarium* pathogens, including Bacillomycin D, surfactin, and fengycin (synonymous with plipastatin)^215^. Bacillomycin D and fengycin can inhibit the growth of hyphae, cause morphological alterations or destruction of cell walls and plasma membranes, and trigger the cell lysis of *F. graminearum* and *Fusarium moniliforme* through the accumulation of ROS. Moreover, Iturin A, a kind of Bacillomycin D, can also control the T‐2 toxin synthesis of *F. oxysporum* by inhibiting *TRI5* expression^216^. Regarding *F. moniliforme*, surfactin can inhibit hyphal growth and induce ROS, damaging DNA and protein in living cells^217^. In addition to direct interactions, bacterial BCAs can also protect plants against *Fusarium* pathogens in indirect ways. Chen et al.^218^ suggested that *B. velezensis* LM2303 has various biocontrol mechanisms against *F. graminearum*, such as enhancing wheat systemic resistance, facilitating wheat plant growth, and outcompeting for space and nutrient competition via efficient colonization and antibacterial metabolites.

The genera *Aureobasidium, Cladosporium, Clonostachys, Cryptococcus, Sarocladium*, and *Trichoderma* are the representative fungal BCAs for *Fusarium* disease management. *Trichoderma* is a notable example of a fungal antagonist, as it not only inhibits the growth and reproduction of *Fusarium* fungi through mycoparasitism, antibiotics, promotion of plant growth, and the induction of defense responses in host plants, but also has the ability to control the biosynthesis of *Fusarium* mycotoxins and directly degrade *Fusarium* mycotoxins^219^. Błaszczyk et al.^220^ reported that *Trichoderma atroviride* shows significant inhibitory effects on the biosynthesis of mycotoxins (DON, 3ADON, 15ADON, NIV, ZEA, BEA, and MON) of *Fusarium avenaceum*, *Fusarium cerealis*, *F. culmorum*, *F. graminearum*, and *Fusarium temperatum*. Tian et al.^221^, ^222^ indicated that *Trichoderma* fungi can convert ZEA into ZEA sulfate and ZEL sulfate by sulfation and glycosylate TRIs into glycosylated forms. Galletti et al.^223^ found that *Trichoderma gamsii* B21 can remove FUMs from liquid cultures, but the mechanisms were unclear. Recently, the utilization of endophytic fungi as BCAs has become regarded as a compelling strategy for controlling plant disease. Kemp et al.^224^ found that the wheat endophytic fungus *Sphingobacterium zeae* NRRL 34560 can induce defense responses in wheat, effectively controlling FHB and DON contamination in a greenhouse.

Mycoviruses, also known as fungal viruses, are parasitic viruses that infect a wide range of filamentous fungi and yeasts, including *Fusarium* fungi. Mycoviruses typically do not exert phenotypic effects on their hosts; however, some can induce negative consequences, such as hypovirulence^225^. These hypovirulence‐related mycoviruses have great potential as BCAs. In total, mycoviruses have been documented in 13 *Fusarium* species, including *F. asiaticum, Fusarium boothii, Fusarium circinatum, Fusarium coeruleum, Fusarium globosum, F. graminearum, F. incarnatum, Fusarium langsethiae, F. oxysporum, Fusarium poae, Fusarium pseudograminearum, Fusarium solani, and Fusarium virguliforme*. Most *Fusarium* mycoviruses establish latent infections, but some, including Fusarium graminearum virus 1 (FgV1), Fusarium graminearum virus‐ch9, Fusarium graminearum hypovirus 2 (FgHV2), Fusarium oxysporum f. sp. dianthi mycovirus 1, and Fusarium pseudograminearum megabirnavirus 1 (FpgMBV1), cause hypovirulence^225^. Moreover, FgV1 and FgHV2 infections can reduce DON production in *F. graminearum* ^226^, ^227^. Li et al.^228^ found that FpgMBV1 eliminates DON accumulation by downregulating the expression of TRI biosynthetic genes. However, most research in this area is still in the laboratory stage and has not been applied in the field.

After harvest, beneficial microorganisms can be used to control *Fusarium* mycotoxin contamination. Lactic acid bacteria (LAB), which are frequently used as biological preservatives, not only can prevent *Fusarium* fungal growth but can also control mycotoxin contamination in food and feed. Strains of *Lactobacillus*, *Bifidobacterium*, *Lactococcus*, *Leuconostoc*, *Pediococcus*, *Propionibacterium*, and *Streptococcus* have been used to control mycotoxin contamination^229^. LAB produce many antifungal metabolites, such as organic acids, hydrogen peroxide, diacetyl, rutherin, reutericyclin, acetoin, bacteriocins, and bacteriocin‐like inhibitory substances^230^. In addition to its antifungal activity, LAB possess the ability to adsorb, degrade, or detoxify *Fusarium* mycotoxins. On one hand, LAB can assimilate mycotoxins toward their cell wall operative groups; on the other hand, LAB can degrade mycotoxins via metabolic apparatus or enzymes^229^.

A drop in the utilization of synthetic preservation has occurred due to its side effects, such as resistance development in pests, nonbiodegradable characteristics, and toxic effects on nontargeted organisms. Therefore, as green preservatives, plant EOs have become widely used substitutes for synthetic preservatives. In the past decade, cinnamon, clove, eucalyptus, fennel, oregano, rosemary, palmarosa, and thyme were the most frequently used EOs to control mycotoxigenic fungi and their mycotoxins. EOs can prevent fungal infection and mycotoxin biosynthesis in many ways, such as the inhibition of fungal growth, destruction of cell permeability, disruption of the electron transport chain, and modulation of gene expression patterns and metabolic processes^231^. Due to the development of nanotechnology, nanoencapsulation of EOs has emerged as a novel strategy to increase the stability of EO constituents, extend their applicability, and overcome their major limitations by controlled release. Singh et al.^232^ fabricated clove oil nanoemulsions and found that they can control the growth of *F. proliferatum* and reduce the FB_1_ and FB_2_ contents of maize.

### Physical method

Physical methods can be used to eliminate mycotoxins during postharvest storage and processing; these include separation, debranning and milling, heat treatment, radiation, and adsorption. Mycotoxins are mainly found in the moldy, fragmented, and discolored parts of grains, and the specific gravity of mycotoxin‐contaminated cereals is lower than that of uncontaminated cereals^233^. These characteristics enable *Fusarium*‐damaged grains to be segregated by aspiration, image processing techniques, gravity separation, and photoelectric separation. In general, the external layer of grains tends to show higher levels of mycotoxin contamination. Partial debranning of 10% of the wheat grain weight can result in a 64% reduction in DON content^234^. Therefore, debranning and milling procedures are important for the decontamination of *Fusarium* mycotoxins. After polishing, the *Fusarium* mycotoxin content of white rice can be markedly decreased^235^. Regarding the milling process, *Fusarium* mycotoxins tend to accumulate in the outer fractions of wheat grains (bran, flour shorts, screenings, and middlings), which are primarily utilized as animal feeds, and lower concentrations are found in the inner fractions (flour or semolina) intended for human consumption^236^.

There are a series of innovative physical methods to control *Fusarium* mycotoxin contamination that are usually based on nonthermal techniques: cold atmospheric plasma (CAP), electron beam irradiation, pulsed light, and so on. The CAP process makes use of an ionized gas at near room temperature to inactivate microorganisms^237^. CAP is also successful at degrading mycotoxins due to the oxidizing potential of plasma. Compared to conventional and other nonthermal approaches, CAP shows rapid efficacy in controlling fungi and mycotoxins, exerts minimal influence on product quality, and requires only low energy consumption^238^. Feizollahi et al.^239^ found that CAP treatment can reduce DON in barley by 49% in 6 min, and Wielogorska et al.^240^ reported a significant decrease (66%) in FB_1_ in maize following a 10‐min treatment with CAP. So, CAP technology represents remarkable progress in the food industry. Unfortunately, prior studies have only been successful on the laboratory scale. Therefore, additional research is warranted to demonstrate the effectiveness within wide‐scale food production and different food matrices, as well as of different types of gasses^233^.

## CONCLUDING REMARKS

In this review, we elucidate the effects of *Fusarium* mycotoxin contamination on food safety and summarize the countermeasures to prevent or mitigate harm to humans. In addition, we discuss how CC influences *Fusarium* mycotoxin contamination—many models have been constructed on historical or current datasets of climatic conditions to anticipate the interactions of *Fusarium* mycotoxins with CC. However, most models lack the [CO_2_] factor, which exerts a significant influence on the growth of both crops and fungi. The ultimate goal of controlling *Fusarium* mycotoxin contamination is to protect human health. Nevertheless, few studies have estimated the transfer of mycotoxins from fields to human foods or their eventual impact on human health under different CC scenarios.

*Fusarium* mycotoxins cannot be completely eliminated worldwide. All we can do is to reduce contamination to acceptable levels that do not threaten human health. Therefore, a number of detection technologies and management strategies have been developed. Both agricultural production and industrial food processing are economic activities; therefore, scholars and practitioners should consider both the economic benefits and the costs of the application of *Fusarium* mycotoxin countermeasures to producers. With the improvement of living standards, environmental stability begins to take precedence over production efficiency and economic benefits. The development and recommended use of farm chemicals is a representative example. So, the development of *Fusarium* mycotoxin countermeasures should be based on a balance among these three factors. Good agricultural practices (GAPs) are a set of guidelines for producing safe and healthy food and nonfood agricultural products through on‐farm and post‐production practices, with due consideration of the sustainability of economic, social, and environmental aspects^241^. With regard to the control of *Fusarium* mycotoxins, GAP is of great significance and has been applied in many countries^242^. Nevertheless, it is not easy to change the agricultural practices of smallholder farmers. These changes may require more communication, education, and support from local governments, especially in developing countries.
